# Isolation and Characterization of Protein Tyrosine Phosphatase 1B (PTP1B) Inhibitory Polyphenolic Compounds From *Dodonaea viscosa* and Their Kinetic Analysis

**DOI:** 10.3389/fchem.2018.00040

**Published:** 2018-03-01

**Authors:** Zia Uddin, Yeong Hun Song, Mahboob Ullah, Zuopeng Li, Jeong Yoon Kim, Ki Hun Park

**Affiliations:** Division of Applied Life Science (BK21 Plus), IALS, Gyeongsang National University, Jinju, South Korea

**Keywords:** polyphenolic compounds, *Dodonaea viscosa*, diabetes mellitus, PTP1B inhibition, enzyme kinetics

## Abstract

Diabetes mellitus is one of a major worldwide concerns, regulated by either defects in secretion or action of insulin, or both. Insulin signaling down-regulation has been related with over activity of protein tyrosine phosphatase 1B (PTP1B) enzyme, which has been a promising target for the treatment of diabetes mellitus. Herein, activity guided separation of methanol extract (95%) of *Dodonaea viscosa* aerial parts afforded nine (**1**-**9**) polyphenolic compounds, all of them were identified through spectroscopic data including 2D NMR and HREIMS. Subsequently, their PTP1B inhibitory potentials were evaluated, in which all of the isolates exhibited significant dose-dependent inhibition with IC_50_ 13.5–57.9 μM. Among them, viscosol (**4**) was found to be the most potent compound having IC_50_ 13.5 μM. In order to unveil the mechanistic behavior, detailed kinetic study was carried out, in which compound **4** was observed as a reversible, and mixed type I inhibitor of PTP1B with inhibitory constant (*K*_i_) value of 4.6 μM. Furthermore, we annotated the major metabolites through HPLC-DAD-ESI/MS analysis, in which compounds **3**, **6**, **7**, and **9** were found to be the most abundant metabolites in *D. viscosa* extract.

## Introduction

The globally increasing incidence of diabetes mellitus (DM) has attracted great attention for search of food and herbal remedies having curing and preventive properties. Recent data shows that about 380 million people are suffering from diabetes worldwide, in which more than 90% are type 2 diabetes mellitus (T2DM) (Nguyen et al., 2013; Vo et al., 2015). Type 2 diabetes mellitus is characterized by high blood glucose level, mostly due to insulin resistance which further leads to several complications and badly affects the vital organs (Xue et al., 2007). There are several ways of controlling the hyperglycemia, in which inhibition of PTP1B is one of a well-established therapeutic strategy, which mitigates insulin resistance (Montalibet and Kennedy, 2005). Protein tyrosine phosphatase 1B is one of the important enzyme of PTPs family, which is highly expressed in important tissues such as liver, muscle, and fat which are the specific targets of insulin (Johnson et al., 2002). The prime function of PTP1B is the regulation of insulin and leptin signaling, both of which are important hormones playing their roles in the regulation of cellular metabolism and glucose homeostasis. This enzyme catalyzes the de-phosphorylation of activated insulin receptor and insulin receptor substrate, and results in down-regulation of insulin signaling (Zhang and Zhang, 2007). Additionally, PTP1B also dephosphorylates Janus Kinase 2, thus negatively regulates the leptin signaling pathway and contributes to obesity and metabolic disorders. Similarly, it has been reported that PTP1B deficient mices exhibits high insulin sensitivity and resistance to obesity (Nieto-Vazquez et al., 2007). Thus, the insulin resistance which is produced as a result of PTP1B over activity leads to hyperglycemia and metabolic disorder, which are the main causes of T2DM and obesity (Saltiel and Kahn, 2001). Hence, PTP1B is an effective target and its inhibition has been suggested as a great approach for the treatment of T2DM and prevention of obesity (Montalibet and Kennedy, 2005). Therefore, exploration of bioactive compounds from natural sources having potential to inhibit PTP1B enzyme can play their role in suppression of T2DM and obesity.

*Dodonaea viscosa* (L.) Jacq is an ever green medicinal plant, locally known as “*Ghawraskay*” and “*pribet*” which belongs to Sapindaceae family, and is widely distributed throughout tropical and subtropical countries such as Africa, Mexico, and temperate region of Australia, India, and Pakistan (Lawal and Yunusa, 2013; Khurram et al., 2015). The aerial parts of this plant have been used in traditional Ayurveda system of medicine to heal simple ulcer, rheumatoid arthritis, body wounds, stomach pain, bacterial, and fungal infections (Chhabra et al., 1991; Lawal and Yunusa, 2013). Similarly, in traditional system of medicine the decoction of fresh plant material have been consumed to ameliorate diabetes and suppress hyperglycemia (Arulselvan et al., 2014; Yaseen et al., 2015). Several phytochemical studies have reported that flavonoids, triterpenoids, diterpenoids, and saponines are the major constituents of this plant (Zhang et al., 2012). Moreover, recently our research group has also reported a novel flavonol from this plant (Uddin et al., 2017). Importantly, since ancient times the areal parts of *D. viscosa* have been utilized to treat diabetes and hyperglycemia in several parts of South Asia (Arulselvan et al., 2014; Yaseen et al., 2015). Whereas, the crude extracts have also shown hypoglycemic effects, reported by several experimental studies (Veerapur et al., 2010; Akhtar et al., 2011; Muthukumran et al., 2011). All these antidiabetic and hypoglycemic effects of *D. viscosa* extracts may arise from insulin stimulation due to the downregulation of PTP1B activity. Thus, the above description have pointed out that *D. viscosa* extracts possess significant anti-diabetic potential, however to date no one has explored and thoroughly characterized these individual constituents as antidiabetic agents. So our conjecture is, *D. viscosa* is a rich source of PTP1B inhibitors, which are responsible for the given antidiabetic and hypoglycemic activities.

The current study was aimed to explore the antidiabetic potential of individually purified polyphenolic compounds present in *D. viscosa* extract. For this purpose, phytochemical investigation of its methanol extract was performed to isolate and identify the bioactive compounds responsible for PTP1B inhibition which further leads to hypoglycemic effect. Nine (**1**-**9**) flavonols were isolated and thoroughly characterized by spectroscopic data. All purified compounds were assessed for their PTP1B inhibitory potential. Subsequently, detailed kinetic study of isolated compounds was carried out, which unveiled the inhibitory modes and mechanism of action of these inhibitors. Furthermore, the annotation of each peak in the methanol extract was performed by HPLC-DAD-ESI/MS analysis.

## Materials and methods

### Instruments and chemicals

Bruker AM 500 nuclear magnetic resonance (^1^H NMR at 500MHz, ^13^C NMR at 125 MHz) spectrometer (Bruker, Karlsruhe, Germany) was used for 1D ^1^H and ^13^C NMR, as well as 2D NMR analysis, using CD_3_OD, CDCl_3_, and MeOD with TMS as internal standard (Andover, MA, USA). JEOL JMS-700 mass spectrometer (JEOL, Tokyo, Japan), was applied to get electron ionization mass (EIMS), high resolution electron ionization mass (HR-EIMS), and HR-FABMS. Separation and purification was carried out on medium pressure liquid chromatography (MPLC) instrument (Teledyne Isco, Lincoln, USA), using silica gel and reversed-phase silica gel (C18) cartridges. Thin layer chromatography (TLC) plates which were pre-coated with silica gel 60 F254 (0.25 mm, normal phase, Merck) were used TLC analysis. These TLC plates were visualized in a UVGL-58 254 nm hand-held UV lamp (UVP, Cambridge, UK) or by spraying with 10% H_2_SO_4_ in ethanol followed by heating. SpectraMax M3 Multi-Mode Microplate Reader (Molecular device, USA) was used for enzymatic assays. RP-18 (ODS-A, 12 nm, S-150 μM, YMC), Sephadex LH-20 (Pharmacia Biotech AB, Uppsala, Sweden), Diaion HP-20 and Silica gel (230–400 mesh, Merck), were used for column chromatography. First grade organic solvents were used for isolation and purification. Whereas, analytical grade acetonitrile and water were purchased from J.T. Baker (Phillipsburg, NJ, USA) and used for LCMS analysis. HPLC–DAD–MS analysis were carried out with Agilent (USA) 1100 series system, and ion trap mass spectrometer having ESI interface (Applied Biosystems, Forster, CA, USA).

### Plant material

Previously collected *D. viscosa* aerial parts, at Malakand, Pakistan, in 2014. The (SWAT00261), voucher specimens were kept for future references at University of Swat, KPK, Pakistan. The specie was recognized by Professor Zahid Ullah, university of Swat.

### Preparation of sample

The aerial parts of *D. viscosa* were crushed into powder. Sample (2.0 g) was extracted in methanol (40 mL) for 2 h using sonicator at room temperature. Supernatant liquid was centrifuged at 3,000 g for 6 min. Finally the supernatant was filtered through a 0.45 mm syringe filter and then analyzed by LC-ESI-MS.

### LC-DAD-ESI/MS analysis

HPLC-DAD analysis was performed with 1100 series liquid chromatography (LC) system, equipped with a G1312A pump, G1322A degasser, G1316A oven, and G1313A auto sampler (Agilent Technologies, Palo Alto, CA). The Zorbax Bonus-RP-C18 column (4.6 × 150 mm, 5 mm, Agilent Technologies, Rising Sun, MD) was used for chromatographic seperation. The solvent system consisted of (A) acetic acid/water (0.1/100, v/v), and (B) Acetonitrile (100%) with gradient elution: 0–5 min, B: 15%; 5–11 min, B:15–60%; 11–21 min, B:60–68%; 21–25 min, B:68– 85%, 25–37 min, B:85– 100%; 37–45 min, B:100%, whereas solvent flow rate was 0.5 mL/min, and 30°C was set as optimum temperature for the operations. The UV spectra were measured from 200 to 400 nm, while the final chromatogram was obtained at 270 nm. The elution from LC was allowed to enter the ESI interface without splitting. Mass spectrometric analysis was executed with 3200 Q TRAP LC/MS/MS system (Applied Biosystems, Foster City, CA) having a Turbo Ion Spray probe (450°C) and a Turbo VTM source. Mass spectrometer was run in both, positive and negative ion modes. Analyst software (version 1.4.2) were utilized for data acquisition and processing. Nitrogen gas, being used as nebulizing and drying gas, whose pressure was maintained 60 psi. Capillary voltage 5.5/−4.5 kV, and 450°C source temperature were maintained for optimal functions. Mass spectra were obtained over a range between 100 and 700 *m/z*.

### Extraction and isolation

*D. viscosa* aerial parts (1.3 kg) were chopped in methanol (3 × 14 L) at room temperature for 10 days. Then, under reduced pressure the filtrate was concentrated, giving greenish black gum (185 g). Aerial parts contained a lot of chlorophyll, which were removed by Diaion HP-20 column eluting with 100% MeOH and acetone, successively. The extract (40 g) was chromatographed over silica gel (10 × 40 cm, 230–400 mesh, 950 g), having gradient elution of hexane in ethylacetate (100–0%), that gave 12 different fractions (A-L). Two fractions (B and C, 8.2 g) were combined and separated on silica gel CC (5 × 40 cm, 600 g) using hexane in ethylacetate (100–0%), giving 15 sub fractions (A1-A15). Further, sub-fractions (A6-A10, 1.6 g) enriched with compounds **1**, **2**, and **4** were subjected to silica gel CC, and eluted with gradient flow of hexane to ethylacetate (50:1 → 1:1) which afforded **1** (22 mg), **2** (19 mg), and **4** (31 mg). Sub fractions (A11-A14, 1.05 g) possessing compounds **3**, and **5** were chromatographed over silica gel CC, having mobile phase hexane to ethylacetate (30:1 → 1:1) giving compound **3** (122 mg), and **5** (13 mg). Fractions (E-G, 4.1 g) containing compounds **6** and **7** were separated on silica gel CC using dichloromethane (CH_2_Cl_2_) and acetone (60:1 → 5:1) yielding compound **6** (56 mg) and **7** (85 mg). Finally fractions (I-K, 3.2 g) having compounds **8** and **9** were subjected to MPLC using C18 column and eluted with MeOH in H_2_O (0–100%), it resulted in two main separated fractions consisting of each individual compound. Further each fraction was continuously chromatographed over sephadex LH-20 column, MeOH-H_2_O (90:10) until collecting pure compound **8** (9 mg) and **9** (61 mg).

### Protein tyrosine phosphatase 1B (PTP1B) inhibitory activity

Protein tyrosine phosphatase 1B inhibitory assay was performed with slight modification to the previously reported method (Song et al., 2017). Tris–HCl buffer (25 mM, pH 7.5) which contained 1 mM (ethylenediaminetetraacetic acid) EDTA, 1 mM dithiothreitol (DTT), and 2 mM β-mercaptoethanol was used as buffer solution, whereas *p*-nitrophenyl phosphate (*p*NPP), a substrate of PTP1B was utilized for enzyme activity measurement. Enzyme assay was started by mixing 10 μL of test compounds with 130 μL of Tris-HCl buffer solution in 96-well plate, then 20 μL of enzyme (EC 3.1.3.48, 1μg/mL), and finally 40 μL of substrate (4 mM *p*NPP) was added at 37°C for 10 min. During the substrate hydrolysis, *p*NPP was de-phosphorylated, giving product *p*NP, which was detected by Spectra Max M3 Multi-Mode Microplate reader (Molecular devices, USA) at a wavelength of 405 nm for 30 min. The concentration of tested compounds which inhibited 50% of enzyme activity were designated as the IC_50_ values. Kinetic parameters were determined by Line weaver-Burk and Dixon plots. Sigma Plot (SPCC Inc., Chicago, IL, USA) was utilized for data setting and calculation.

### Protein tyrosine phosphatase 1B (PTP1B) inhibitory kinetics

In order to study detailed kinetics of PTP1B, Michaelis-Menten equation (4) is applied, who's reciprocal is taken which transform to a new equation, Line weaver-Burk equation (5). Using this equation Line weaver-Burk plots were constructed, which were applied for the analysis of inhibitory kinetics of test compounds. Different inhibitor concentrations accompanied with varying substrate concentrations were used for determination of enzymes steady-state rates, subsequently kinetic parameters related to PTP1B inhibition were also determined. The values of inhibitory constants *K*_I_ and *K*_IS_, when free enzyme binds with inhibitor (*K*_I_) or enzyme-substrate-complex interact with inhibitor (*K*_IS_) were obtained from secondary plots of the slopes and vertical intercept (1/V^app^max) vs. inhibitors concentration. The representative Equations (1–3) were used to acquire *K*_I_ and *K*_IS_ values (Chiari et al., 2011).

(1) $$1/V\;=\;K_{\text{m}}/V_{\text{max}}(1+[\text{I}]/K_{\text{I}})1/\text{S }+\;1/V_{\text{max}})$$

(2) $$\text{Slope }=\;K_{\text{m}}/K_{\text{I}}V_{\text{max}}[\text{I}]+K_{\text{m}}/V_{\text{max}}$$

(3) $$\text{Intercept }=\;1/K_{\text{IS}}V_{\text{max}}[\text{I}]+1/V_{\text{max}}$$

For inhibition parameters, an assay was performed having different substrate concentrations (0.437–1.75 mM), and several dilutions of tested compounds.

(4) $$V\;=\;V_{\text{max}}[\text{S}]/K_{\text{m}}+[\text{S}]$$

(5) $$1/V\;=\;K_{\text{m}}/V_{\text{max}}\times 1/[\text{S}]+1/V_{\text{max}}$$

## Results

### LC-DAD-ESI/MS analysis

Comparative analysis of the bioactive compounds with in the areal parts of *D. viscosa* were carried out using LC-DAD-ESI/MS. As presented in Figure **5**, the major and minor peaks of phenolic metabolites were separated chromatographically with in the given time (45 min). The absorbance was measured at 270 nm which was selected as the most sensitive wavelength among other measured wavelengths. The detailed characteristics of each metabolite such as retention time, wavelength, molecular ion, elemental composition, and product ions are shown in Table 2, which were used for the identification of these phenolic metabolites by comparing with the isolated standard compounds and the previously reported spectroscopic data (Sachdev and Kulshreshtha, 1986; Muhammad et al., 2012, 2015; Wabo et al., 2012). All peaks of *D. viscosa* extract showed molecular ions having masses which were consistent with our isolated compounds [M + H]^+^ at *m/z* 345.4 (**1**), *m/z* 345.1 (**2**), *m/z* 331.4 (**3**), *m/z* 413.3 (**4**), *m/z* 429.4 (**5**), *m/z* 431.5 (**6**), *m/z* 417.4 (**7**), *m/z* 595.1 (**8**), and *m/z* 625.2 (**9**).

In order to confirm the structures of detected metabolites in methanol extract, fragmentation analysis was performed and carefully analyzed. For example, the most potent compound representing peak **4** (t_R_ = 30.0 min) having molecular ion [M + H]^+^ at *m/z* 413.3, showed fragment ions *m/z* 382.7 and *m/z* 285.3, respectively. Both fragment ions are the diagnostic products, which are consistent with our results of compound **4** (Molecular formula C_23_H_24_O_7_, [M^+^] ion at *m/z* 412.1520). In view of the given analysis we designated peak (**4**) as 5,7-dihydroxy-3,6-dimethoxy-2-(4-methoxy-3-(3-methylbut-2-enyl)phenyl)-4*H*-chromen-4-one. Consistent with this assignment, other peaks such as peaks **8** (t_R_ = 11.6 min) and **9** (t_R_ = 12.2 min) were assigned to Kaempferol 3-O-rutinoside and Isorhamnetin−3-O-robinobioside. Similarly, rest of chromatographic peaks were assigned, accordingly.

### Characterization of bioactive compounds

**Figure 1:**
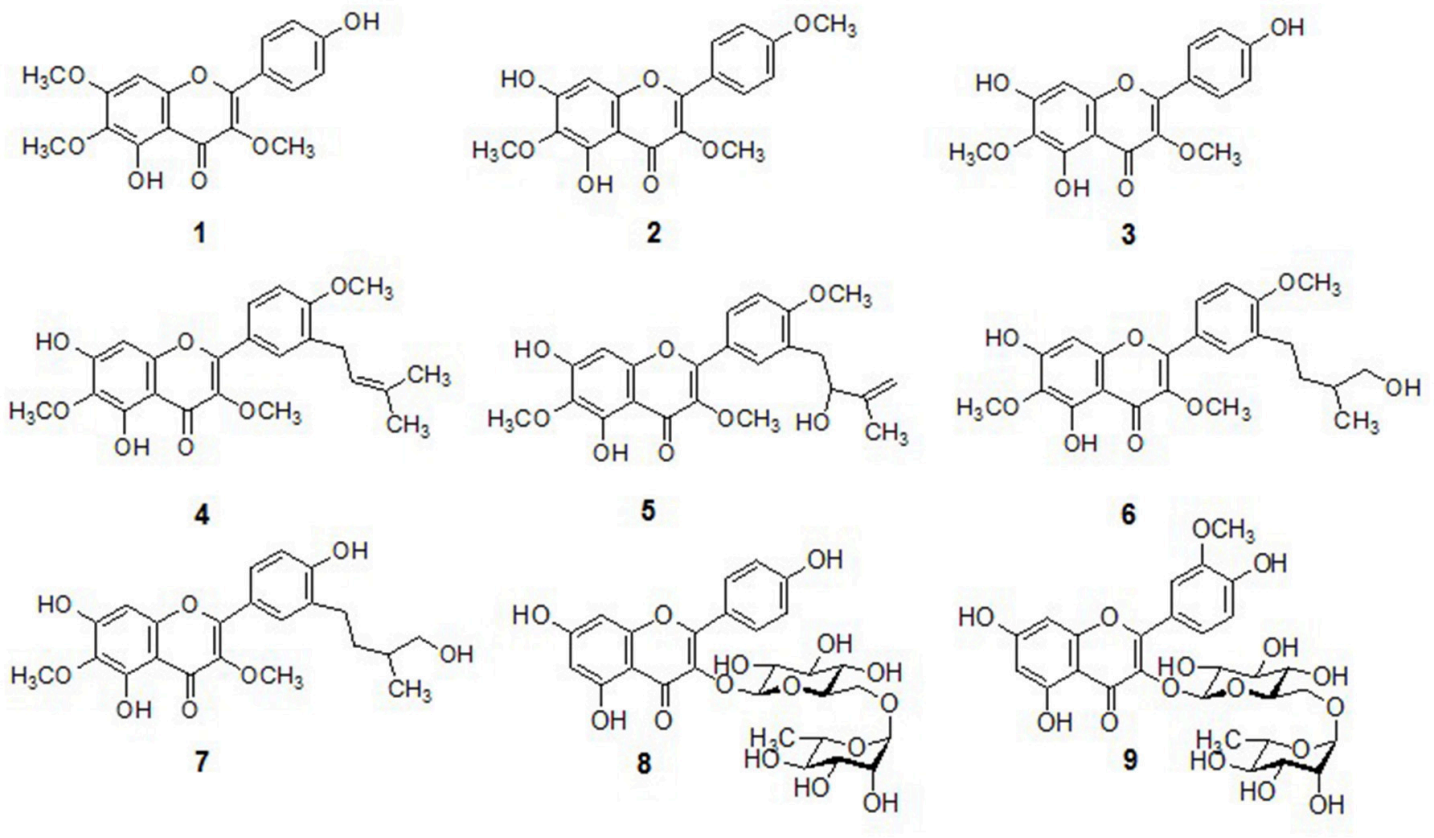
Chemical structures of isolated compounds **1**–**9** from *D. viscosa*.

In preliminary screening, methanol extract of *D. viscosa* was investigated for their ability to inhibit PTP1B activity. During this assessment, methanol extract displayed potent inhibition against PTP1B having IC_50_ value of 32.7 μg/mL, further we proceeded to identify the compounds responsible for the PTP1B inhibitory effects. Purification of the given extract over different reagents such as silica gel, octadecyl-functionalized silica gel (C18), and sephadex LH-20 gave nine (**1**–**9**) polyphenolic compounds. As given in Figure 1, all the isolated compounds were recognized as penduletin (**1**), 5,6-dihydroxy-3,4′,7-trimethoxyflavone (**2**), viscosine (**3**), viscosol (**4**), 5,7-dihydroxy-3′-(2-hydroxy-3-methylbutenyl)-3,6,4′-trimethoxy-flavone (**5**), 5,7-dihydroxy-3′-(3-hydroxy-methylbutyl)-3,6,4′-trimethoxyflavone (**6**), 5,7,4′-trihydroxy-3′-(3-hydroxymethylbutyl)-3,6-dimethoxyflavone (**7**), Kaempferol 3-O-rutinoside (**8**), and Isorhamnetin3-O-robinobioside (**9**), through extensive spectroscopic data consisting of 2D NMR, HREIMS and LC-ESI/MS analysis, as well as comparing with previously reported data (Sachdev and Kulshreshtha, 1986; Muhammad et al., 2012, 2015; Wabo et al., 2012) (Supplementary Materials).

For example, the most active compound (**4**) was isolated as a pale yellow solid, was assigned the molecular formula C_23_H_24_O_7_ by HREIMS (*m/z* 412.1520 [M^+^], calculated: 412.1522). A flavonol skeleton was predicted by ^1^H and ^13^C NMR data, along with DEPT results which highlighted the presence of 23 carbon and 24 proton atoms. A-ring of the skeleton was confirmed by the presence of C6-OCH_3_ which was proved by strong HMBC correlation of OCH_3_ (δ_H_ 4.03) and C-6 (δ_C_ 130.0). Moreover, HMBC correlation of H-8 (δ_H_ 6.54) with oxygenated carbons C-7 (δ_C_ 152.3) and C-8a (δ_C_ 155.2) was observed which clarified more the desired skeleton. After that C-ring substitution of C3-OCH_3_ was confirmed by strong HMBC connectivity of OCH_3_ (δ_H_ 3.83) with C-3 (δ_C_ 138.3). The COSY connectivity between H-2′ (δ_H_ 7.89), H-5′ (δ_H_ 6.93, d, *J* = 8.7 Hz), and H-6′ (δ_H_ 7.93, dd, *J* = 2.3, 8.6 Hz) assisted to identify B-ring of the flavonol. Furthermore, the methoxy moiety C4′-OCH_3_, was located by HMBC correlation of oxygenated carbon C-4′ (δ_C_ 159.6) with OCH_3_ (δ_H_ 3.90) and H-5′/H-6′ (δ_H_ 6.93/7.93) respectively. A characteristic prenyl group attachment at C-3′ was inferred from continuous protons network across H-7′ (δ_H_ 3.36) and H-8′ (δ_H_ 5.33) in COSY spectrum. Furthermore, location of this prenyl functionality was also proved by HMBC correlation of H-7′ (δ_H_ 3.36) with C-2′ (δ_C_ 130.0), C-3′ (δ_C_ 129.4), and C-4′ (δ_C_ 159.4) as well as this correlation was also existed with C-8′ (δ_C_ 121.7) and C-9′ (δ_C_ 133.4). Thus, compound **4** was elucidated as 5,7-dihydroxy-3,6-dimethoxy-2-(4-methoxy-3-(3-methylbut-2-enyl)phenyl)-4*H*-chromen-4-one, also known viscosol, as shown in Figure 1.

### Protein tyrosine phosphatase 1B (PTP1B) inhibitory activity

In order to validate the hypothesis that *D. viscosa* is a rich source of antidiabetic agents, all of the isolated polyphenolic compounds (**1**-**9**) were tested for their inhibitory potentials against PTP1B. Enzyme activity was determined using a previously established protocol with slight changes, in which hydrolysis of *p*-nitrophenyl phosphate was monitored spectrophotometrically (Song et al., 2017). As shown in Table 1, all isolated constituents displayed significant inhibition against the target enzyme with IC_50_ values ranging from 13.5 to 57.9 μM. Among them, compound **4** (IC_50_ = 13.5 μM) exhibited significant inhibition against PTP1B, which seems to be the most potent inhibitor displaying 6 and 2.5-fold more potency than its mother skeletons **1** (IC_50_ = 57.9 μM) and **2** (IC_50_ = 32.2 μM), respectively (Table 1). The higher potency of compound **4** having an attached prenyl group relative to compounds **1** and **2** highlighted the importance of methyl-butenyl moiety. Similarly, compounds **3**, **5**, and **8** also showed strong PTP1B inhibition with IC_50_ values of 18.7, 28.4, and 20.5 μM, respectively. Whereas, compounds **6**, **7**, and **9** expressed moderate activities with IC_50_ values of 56.0, 41.8, and 42.9 μM. Generally, most of the compounds significantly inhibited the target enzyme in dose-dependent manner.

**Table 1:** Inhibitory effects of compounds **1**-**9** on PTP1B activities.

**Compounds**	**Protein tyrosine phosphatase 1B**	
	**IC_50_ (μM)^a^**	**Type of inhibition (*K*_*i*_^b^, μM)**
1	57.9 ± 0.6	Mixed (27.3 ± 0.8)
2	32.2 ± 0.8	Mixed (16.3 ± 0.5)
3	18.7 ± 0.6	Mixed (6.9 ± 0.4)
4	13.5 ± 0.3	Mixed (4.6 ± 0.5)
5	28.4 ± 0.9	Mixed (10.1 ± 0.4)
6	56.0 ± 0.3	Mixed (22.8 ± 0.7)
7	41.8 ± 0.5	Mixed (14.3 ± 0.6)
8	20.5 ± 0.8	Non-competitive (13.0 ± 0.2)
9	42.9 ± 0.4	Non-competitive (26.4 ± 0.8)
Ursolic acid^d^	19.5 ± 1.2	NT^c^

a *All compounds were examined as set of experiments repeated three times; IC_50_ values of compounds represent the concentration that caused 50% enzyme activity loss*.

b *Values of inhibition constant*.

c *NT: not tested*.

d *Positive control*.

**Table 2:** Identification of phenolic metabolites in the areal parts of *D.viscosa* methanol extract by LC-DAD-ESI/MS.

**Peak no**.	**UV λ_max_ (nm)**	**t_R_(min) at 270 nm**	**Detected precursor ion (*m/z*)**	**Molecular formula**	**MS/MS**	**Identification**
1	272, 366	20.3	345.4 [M+H]^+^	C_18_H_16_O_7_	284.5, 312.4	Penduletin
2	270, 349	22.0	345.1 [M+H]^+^	C_18_H_16_O_7_	284.5	5,7-dihydroxy-3,6,4′-trimethoxyflavone
3	268, 348	20.1	331.4 [M+H]^+^	C_17_H_14_O_7_	298.3, 316.6	5,7,4′-trihydroxy-3,6-dimethoxyflavone
4	270, 337	30.0	413.3 [M+H]^+^	C_23_H_24_O_7_	285.3, 382.7	Viscosol
5	272, 338	20.7	429.4 [M+H]^+^	C_23_H_24_O_8_	283.6, 411.5	5,7-dihydroxy-3′-(2-hydroxy-3-methyl 3-butenyl)-3,6,4′-trimethoxyflavone
6	274, 346	22.9	431.5 [M+H]^+^	C_23_H_26_O_8_	357.5	5,7-dihydroxy-3′-(3-hydroxy-methylbutyl)-3,6,4′-trimethoxyflavone
7	272, 345	20.0	417.4 [M+H]^+^	C_22_H_24_O_8_	331.3, 343.2	5,7,4′-trihydroxy-3′-(3hydroxy methylbutyl)-3,6 dimethoxyflavone
8	265, 340	11.6	593.5 [M-H]^−^	C_27_H_30_O_15_	371.2	Kaempferol 3-O-rutinoside
9	265, 340	12.2	623.9 [M-H]^−^	C_28_H_32_O_16_	593.5, 315.1	Isorhamnetin 3-robinobioside

### Protein tyrosine phosphatase 1B (PTP1B) inhibitory kinetics

**Figure 2:**
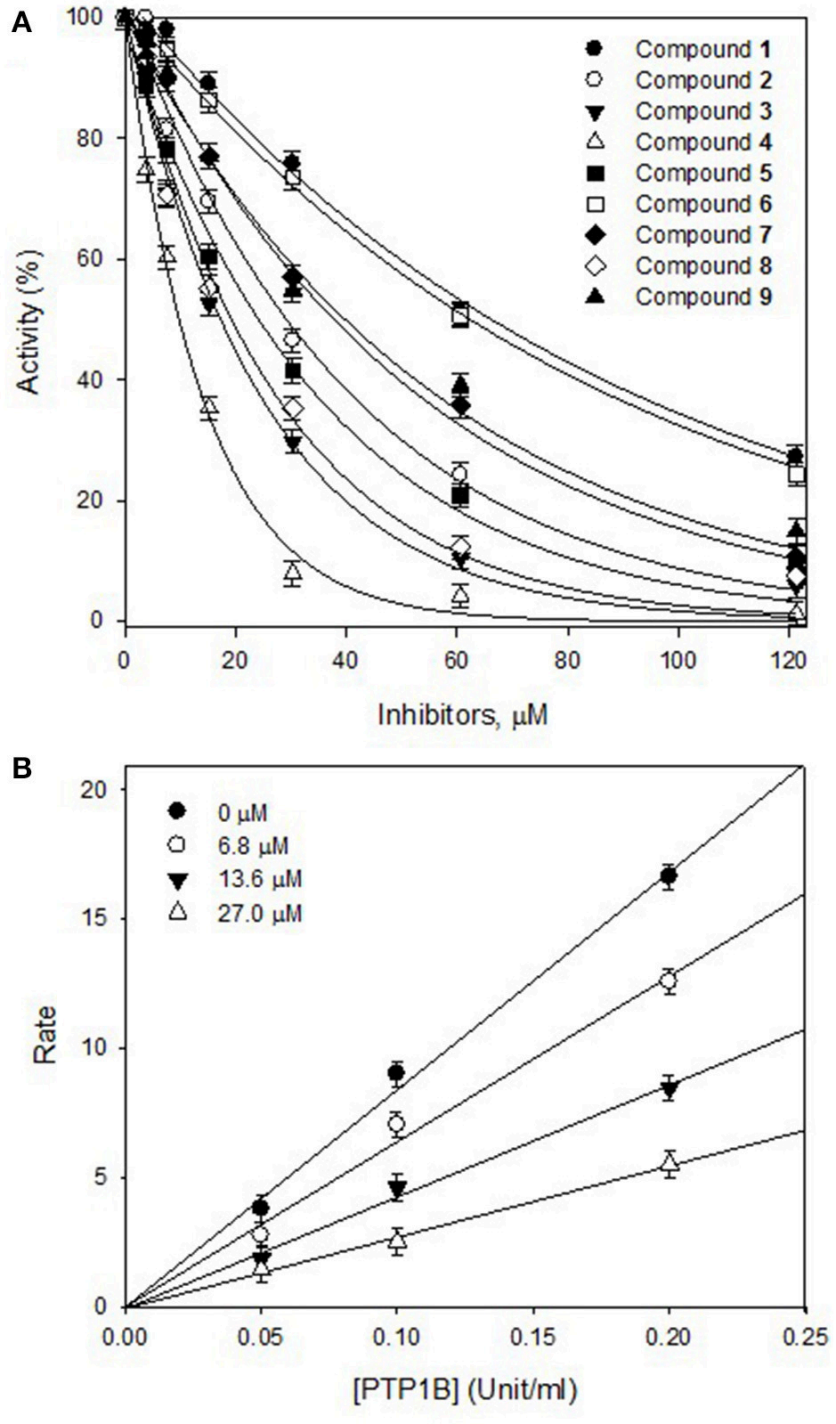
**(A)** Inhibitory effects of purified compounds (**1**-**9**) on the PTP1B catalyzed hydrolysis of *p*-nitrophenyl phosphate (*P*NPP). **(B)** Reversible inhibitory mechanism of the potent compound **4** against PTP1B.

**Figure 3:**
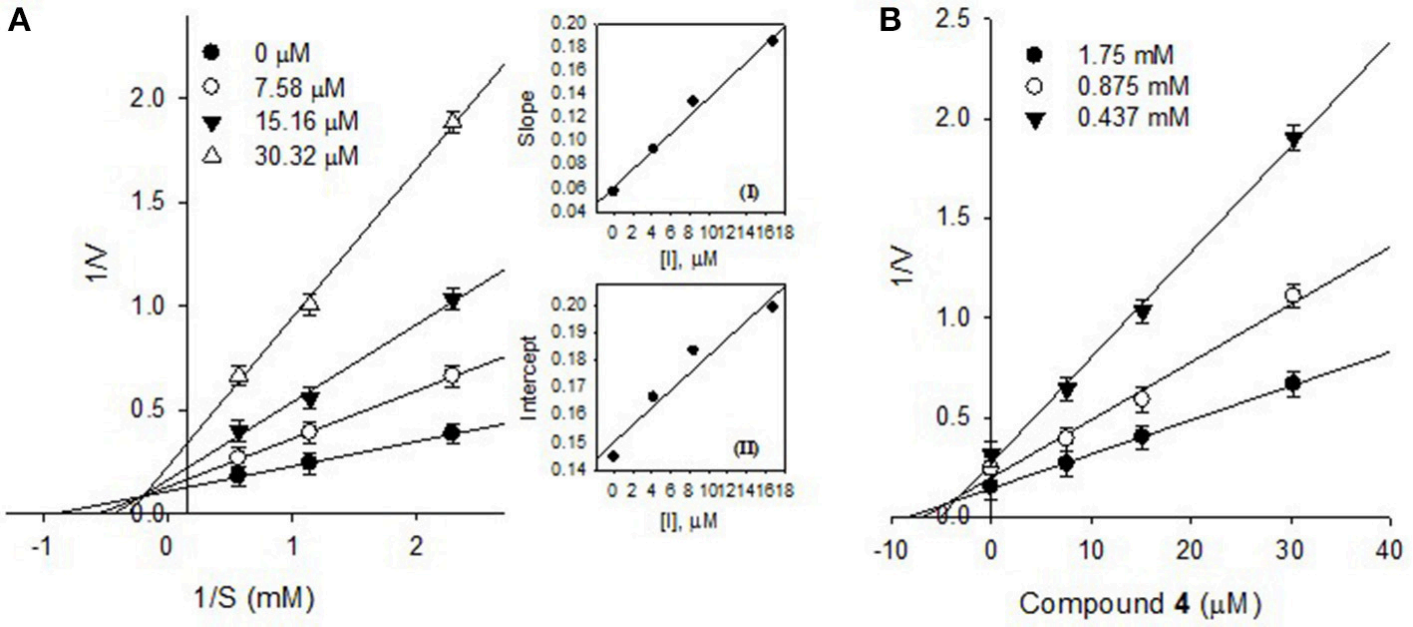
**(A)** Lineweaver-Burk plots for the inhibition of PTP1B by compound **4**, Secondary plots, Inset (I) and (II) represent plots of the slope and the intercept vs. inhibitor (**4**) concentrations. **(B)** Dixon plots for inhibition of PTP1B by compound **4**.

The effects of different concentrations of the bioactive active compounds on the hydrolysis of *p*-nitrophenyl phosphate by PTP1B were studied. We assayed different concentrations (3.75, 7.5, 15, 30, 60, and 120 μM) of inhibitors (**1**-**9**) with the given enzyme and substrate. As a result, we observed a gradual decrease in enzyme activity (% activity) with increasing inhibitors concentrations as shown in Figure 2A. It indicated that these inhibitors decreased PTP1B activity in dose-dependent manner. Representatively, the reversible inhibition displayed by the most potent compound **4** was also plotted (Figure 2B). Plots of residual enzyme activity vs. enzyme concentration at different concentrations (0, 6.7, 13.5, and 27 μM) of compound **4** gave a family of lines which intersected on x-axis at 0 intercept. It is apparent from the Figure 2B, that increasing concentration of compound **4** resulted in reduction of slopes of the lines, all of which intersected at the origin. Similarly, rest of inhibitors demonstrated an identical relationship between enzyme activities and their concerned enzyme concentrations. In order to explore the mechanistic behavior and kinetic features of the tested compounds, detailed kinetic study was performed. For this purpose the target enzyme was assayed in different concentrations of substrate (0.437, 0.875, 1.75 mM), in the presence of several dilutions of the respective compound, been graphically presented in double reciprocal plots (Linweaver-Burk and Dixon plots). Representatively, here we have explained compound **4** in detail, the Lineweaver-Burk plots of (**4**) produced a family of straight lines which intersected on the left side of y-axis and above the x-axis as shown in Figure 3A. The inhibitory constants (*K*_I_ and *K*_IS_) were obtained by plotting the slopes and intercepts vs. different concentrations of compound **4** (Figure 3A, insets). The *K*_I_ and *K*_IS_ parameters were fitted to Equations (2, 3), as a result of which the following values were calculated, *K*_I_ = 4.1 ± 0.2 μM and *K*_IS_ = 26.4 ± 0.4 μM. These results indicated that compound **4** effectively blocked free enzyme as compared to enzyme-substrate complex, and accordingly this mechanism was assigned Mixed type I (Uddin et al., 2016). On other hand, Dixon plots (1/V vs. [I]) were constructed, which gave a family of straight lines passing through the origin on x-axis, as given for a representative compound **4** (Figure 3B), displaying *K*_i_ value of 4.6 μM. In kinetic plots the abscissa [I] is the concentration of compound, where the coordinate1/V represents reciprocal of enzyme activity.

**Figure 4:**
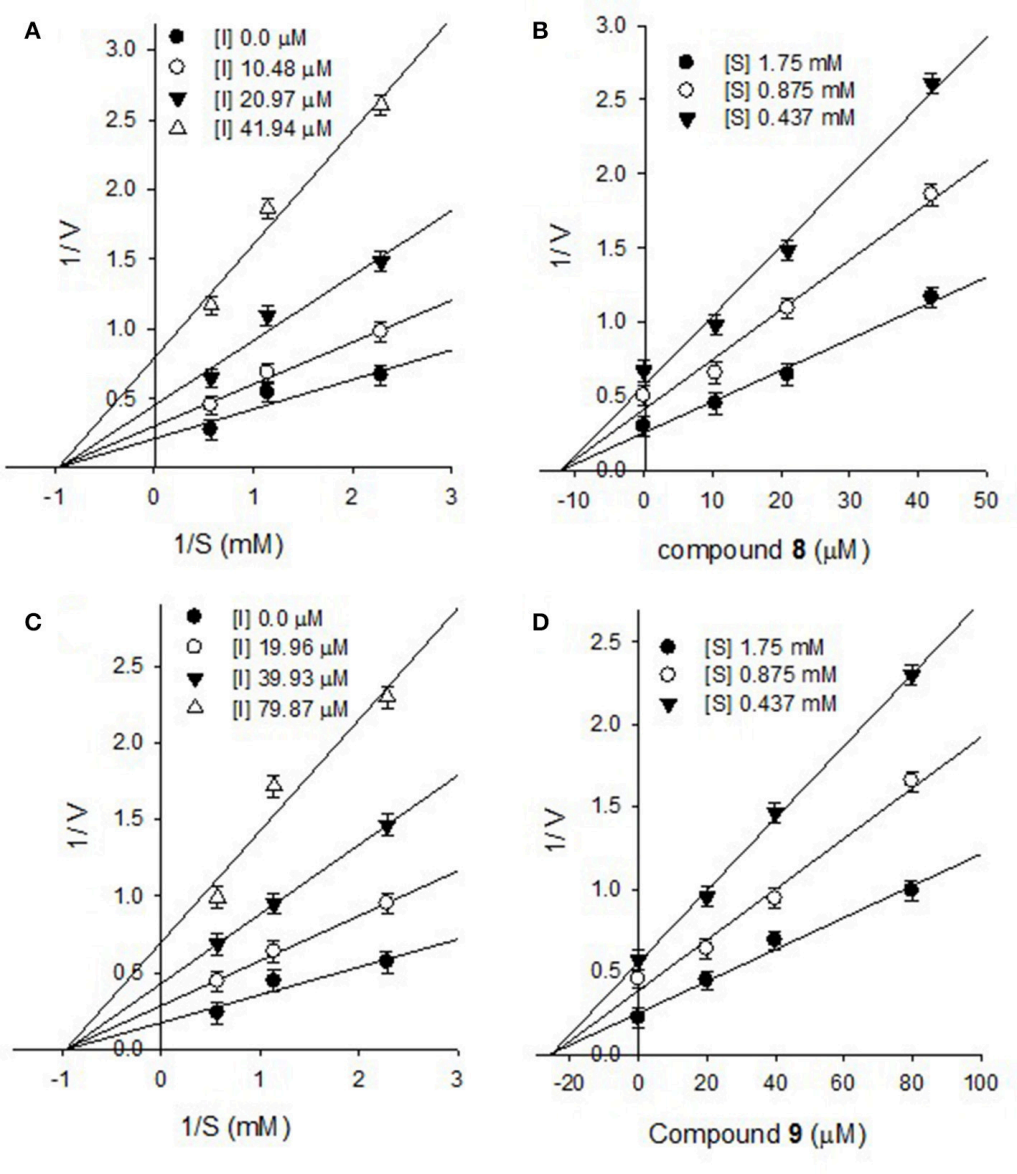
Lineweaver-Burk plots for the inhibition of PTP1B by compound **8 (A)** and compound **9 (C)**. Dixon plots for inhibition of PTP1B by compound **8 (B)** and compound **9 (D)**.

Furthermore, the inhibitory kinetics of the remaining methoxylated flavonols (**1**-**3**, **5**-**7**), which were also analyzed by Line weaver- Burk plots displayed similar pattern (Supplementary Materials). These results demonstrated that all of methoxylated compounds were mixed type inhibitors of PTP1B. Moreover, the glycosylated flavonols (**8** and **9**) exhibited non-competitive inhibitory modes, as illustrated in Figure 4. The Line weaver-Burk plots of **8** and **9**, shown in Figures 4A,C, clearly indicated that the *K*_m_ remained constant while *V*_max_ of the reaction getting changed with increasing inhibitors concentrations. Finally, the inhibition constants (*K*_i_) values of all other compounds were determined by Dixon plots, ranging from 1.01 to 27.3 μM, as given in Table 1.

## Discussion

Diabetes mellitus is one of the major metabolic diseases, having great impact on global health and economy. Especially, the most predominant T2DM which accounts for more than 90% of diabetes is caused by resistance to insulin (Tamrakar et al., 2014). Since ancient times, the local inhabitants of many south Asian and African countries have been using the whole areal parts of *D. viscosa* for the treatment of diabetes and hyperglycemia (Arulselvan et al., 2014; Yaseen et al., 2015). Moreover, several experimental studies have explored the antidiabetic effects of the whole extract, such as a group of researchers have disclosed the antidiabetic potential of *D. viscosa* areal parts using STZ-induced diabetic rats. Similarly, another study has unveiled the significant hypoglycemic effect of methanol extract (250 mg/kg of body weight) of *D. viscosa* leaves on alloxan-induced diabetic rabbits, whereas the antidiabetic potential of this plant *via* glucose tolerance test of normal and diabetic rats have also been reported (Veerapur et al., 2010; Akhtar et al., 2011; Muthukumran et al., 2011). All the previous studies have used the whole crude extract of *D. viscosa*, and no one has explored the antidiabetic potential of these purified individual metabolites (Veerapur et al., 2010; Akhtar et al., 2011). So, we thoroughly characterized these bioactive compounds by spectroscopic analysis such as 2D NMR, HREIMS, LC-DAD-ESI/MS, and for the first time unveiled that phenolic compounds having flavonoid skeleton were the responsible compounds for the mentioned antidiabetic and hypoglycemic effects.

Activity guided fractionation yielded nine (**1**-**9**) polyphenolic compounds, whose structures were clearly established by extensive spectroscopic data. All of isolated compounds displayed dose-dependent inhibition against PTP1B. It means that, decrease in enzyme activity had direct relationship with inhibitors concentrations, which is also evident from Figure 2A. Most of the compounds exhibited significant inhibition (IC_50_, 13.5–57.9 μM) against PTP1B, the enzyme which causes insulin resistance, being a hallmark of T2DM. Among the purified bioactive constituents, compound **4** emerged to be the most active compound (IC_50_ = 13.5 μM) as compared to their mother skeletons (**1** and **2**). It indicated that the higher efficacy of **4** is attributed to the attached methyl-butenyl moiety (prenyl group) at position C-3′ in ring-B of the flavonoid skeleton. Furthermore, detailed kinetic study was undertaken for all tested constituents, in which methoxylated flavonols (**1**-**7**) displayed reversible, mixed type I inhibition. Reversible inhibition is a mode, in which the inhibitor non-covalently interact with enzyme active site and temporary block enzyme activity (Tan et al., 2017). As shown in Figure 3A, with increasing inhibitor concentrations, the apparent *K*_m_ and *V*_max_ of the reaction were getting changed, which indicated a typical mixed type inhibition by compound **4**. In mixed inhibitory kinetics, the inhibitor either binds to free enzyme (E) or enzyme-substrate complex (ES), and subsequently reduce the velocity of enzymatic reaction (Uddin et al., 2016). To further confirm wither inhibitor (**4**) had more affinity toward free enzyme or enzyme-substrate complex, we measured the residual enzyme-substrate complex. Our results demonstrated that compound **4** more efficiently binds with free enzyme as compared to enzyme-substrate complex, and hence displays mixed type I inhibition. Moreover, the glycosylated flavonols (**8** and **9**) exhibited non-competitive inhibitory modes, where both of them can bind to free enzyme and also to enzyme-substrate complex, as a result of this binding, they suppressed PTP1B activity. Overall, the biological effectiveness of these individual inhibitors varied according to their skeletons. There for, on the basis of attached functionalities to the main skeleton, these inhibitors displayed different activities shown in Table 1.

**Figure 5:**
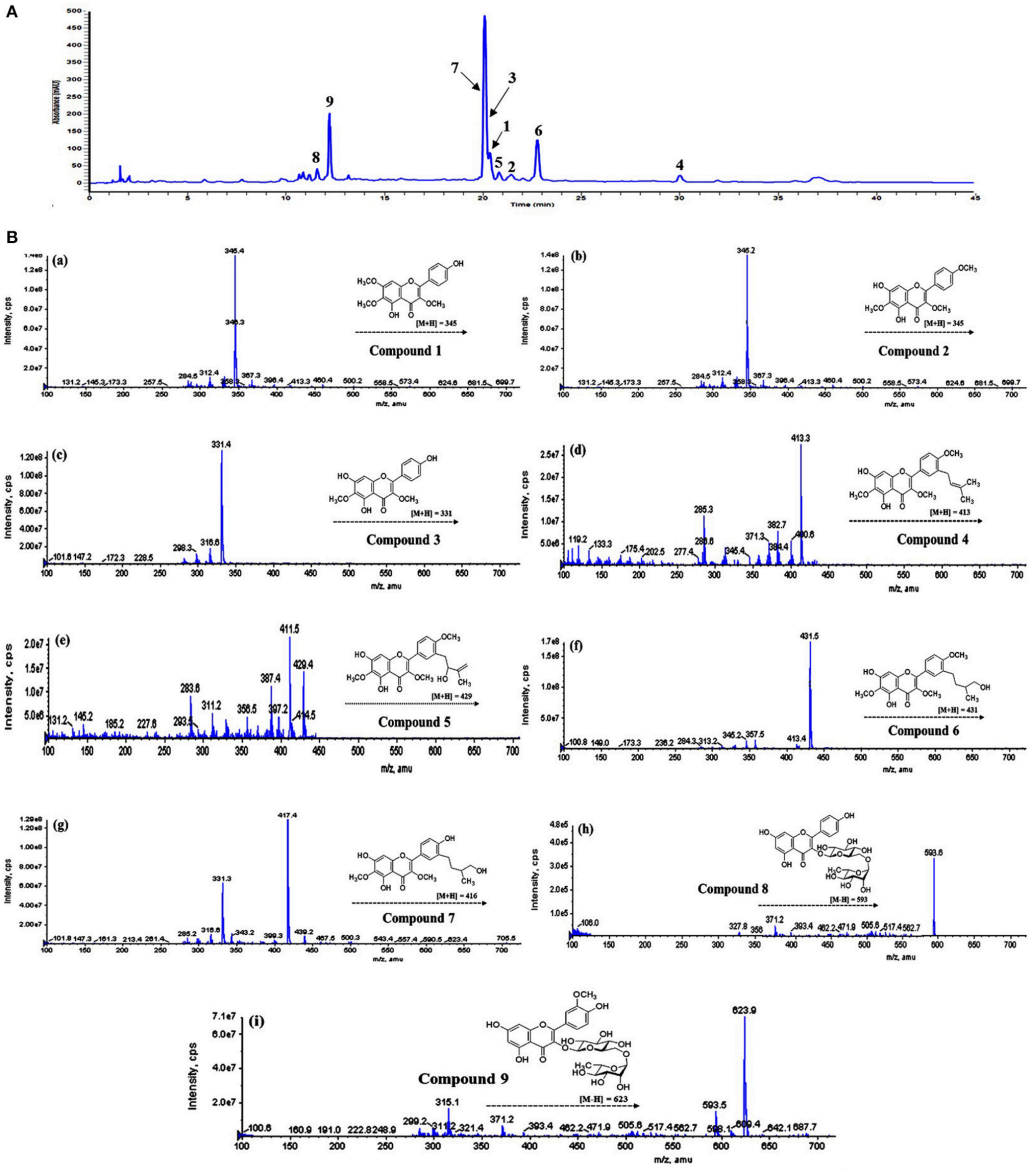
**(A)** HPLC-DAD chromatogram of *D. viscosa* methanol extract detected at 270 nm. **(B)** Mass fragmentation patterns of identified peaks, **(a)** compound **1**, **(b)** compound **2**, **(c)** compound **3**, **(d)** compound **4**, **(e)** compound **5**, **(f)** compound **6**, **(g)** compound **7**, **(h)** compound **8**, **(i)** compound **9**.

We also performed LC-DAD-ESI/MS analysis of the crude methanol extract of *D. viscosa*, and detected the whole major and minor peaks representing each compound in the extract. Interestingly, it is evident from HPLC chromatogram (Figure 5A), that we have completely isolated all the bioactive components present in the methanol extract of *D. viscosa*. Furthermore, characteristic features such as retention times, molecular weights, and product ions of each chromatographic peak were compared with individual purified compound, and successfully annotated each single peak in methanol extract. Based on chromatographic analysis, we observed some major peaks in the crude extract, which were accordingly assumed as the most abundant compounds. The enzyme inhibitory components representing peaks such as **3**, **6**, **7**, and **9** were proven to be the major metabolites in *D. viscosa* extract. As mentioned in the results part, we believe that the phenolic phytochemicals (flavonoids) in the methanol extract have exerted a synergistic effect to inhibit PTP1B enzyme, which are the main responsible compounds to suppress diabetes and hyperglycemia.

In conclusion, the phytochemical investigation of *D. viscosa* afforded nine bioactive flavonols (**1-9**), which significantly inhibited PTP1B enzyme with IC_50_ values ranging from 13.5 to 57.9 μM. Among them, viscosol (**4**) was found to be the most potent inhibitor having IC_50_ of 13.5 μM, and bearing a methyl-butenyl moiety at C-3′ position. Detailed kinetic study assigned compound **4** to be a reversible, and mixed type I inhibitor of PTP1B with *K*_i_ value of 4.6 μM. Furthermore, HPLC-DAD-ESI/MS analysis annotated each peak in which compounds **3**, **6**, **7**, and **9** were the most abundant metabolites in *D. viscosa* extract. Our findings suggests that the antidiabetic potential of *D. viscosa* extract may arise from the collective synergistic effect of the flavonoid compounds by inhibiting PTP1B enzyme. In this regard, further *in-vivo* study is required to validate the inhibitory effects of individual flavonoids and their respective mechanisms using animal model.

## Author contributions

ZU: designed the research work, carried out isolation, purification and structural elucidation of the compounds, and wrote the manuscript; YS and MU: helped with kinetic analysis; JK and ZL: performed statistical calculations and data analysis; KP: who is the corresponding author, helped in project design, and revised the manuscript. All authors reviewed the manuscript and approved for submission.

### Conflict of interest statement

The authors declare that the research was conducted in the absence of any commercial or financial relationships that could be construed as a potential conflict of interest. The reviewer, YL, and handling Editor declared their shared affiliation.
