# Supplementary material for: Isolation and Characterization of Protein Tyrosine Phosphatase 1B (PTP1B) Inhibitory Polyphenolic Compounds From Dodonaea viscosa and Their Kinetic Analysis
Source: Front Chem. 2018 Mar 1;6:40. doi: 10.3389/fchem.2018.00040 (PMC5839231; doi:10.3389/fchem.2018.00040)
Supplement: Supplementary file 1 [file Presentation1.PDF]

SUPPLEMENTARY MATERIAL

Characterization of protein tyrosine phosphatase 1B (PTP1B) inhibitory polyphenolic compounds from *Dodonaea viscosa* and their kinetic analysis

Zia Uddin, Yeong Hun Song, Mahboob Ullah, Zuopeng Li, Jeong Yoon Kim, and Ki Hun Park*

Division of Applied Life Science (BK21 plus), IALS, Gyeongsang National University, Jinju, 660-701, Republic of Korea.

▣ Characterization Data

► ^{1}H-NMR, ^{13}C-NMR and HMBC spectra of other compounds (**1**-**9**).

► EI-Mass, HREI-Mass data of compounds (**1**-**7**).

► ESI-Mass, HRESI-Mass data of compounds (**8**, **9**).

► Enzyme assay, and Kinetic data of compounds (**1**-**9**).

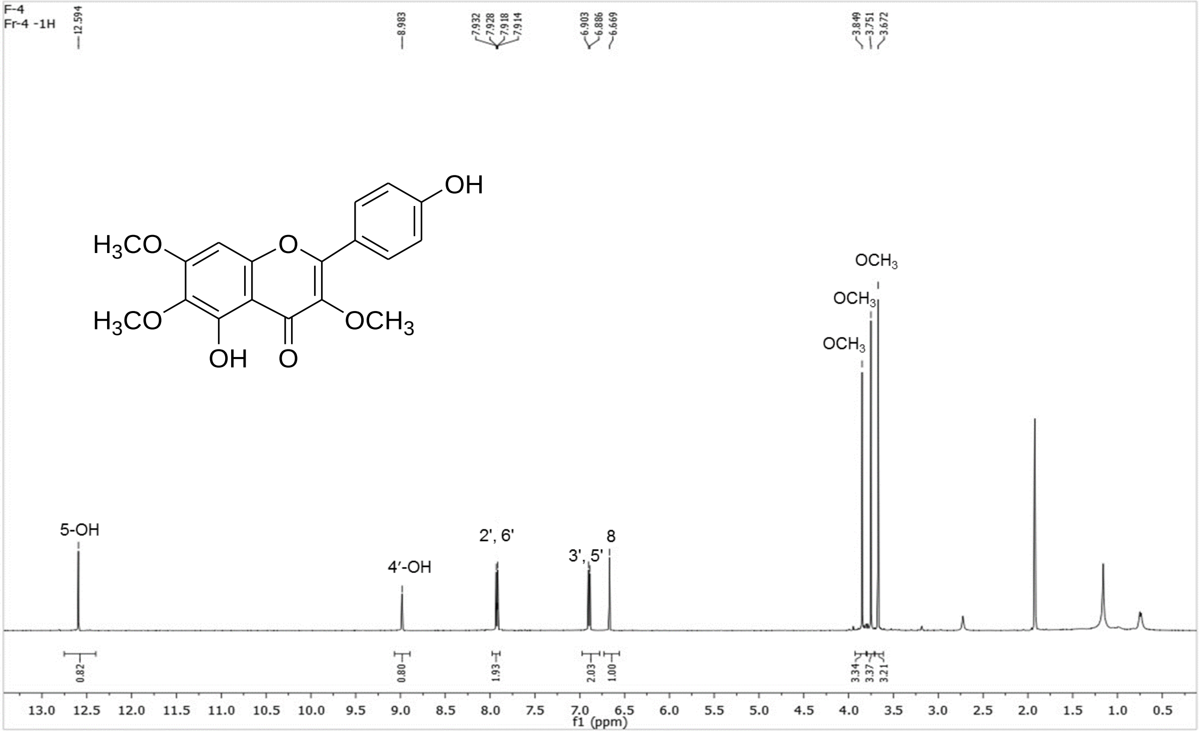

Figure 1. ^{1}H-NMR spectrum of compound **1** (500 MHz, Acetone).

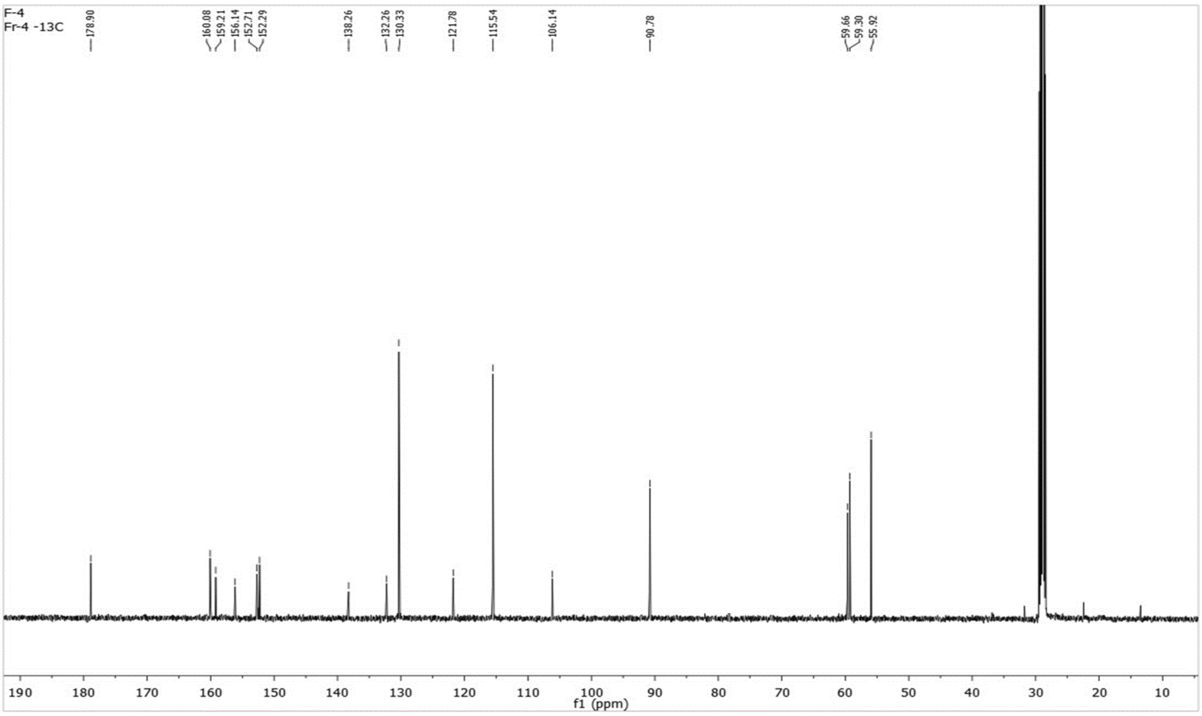

Figure 2. ^{13}C-NMR spectrum of compound **1** (125 MHz, Acetone).

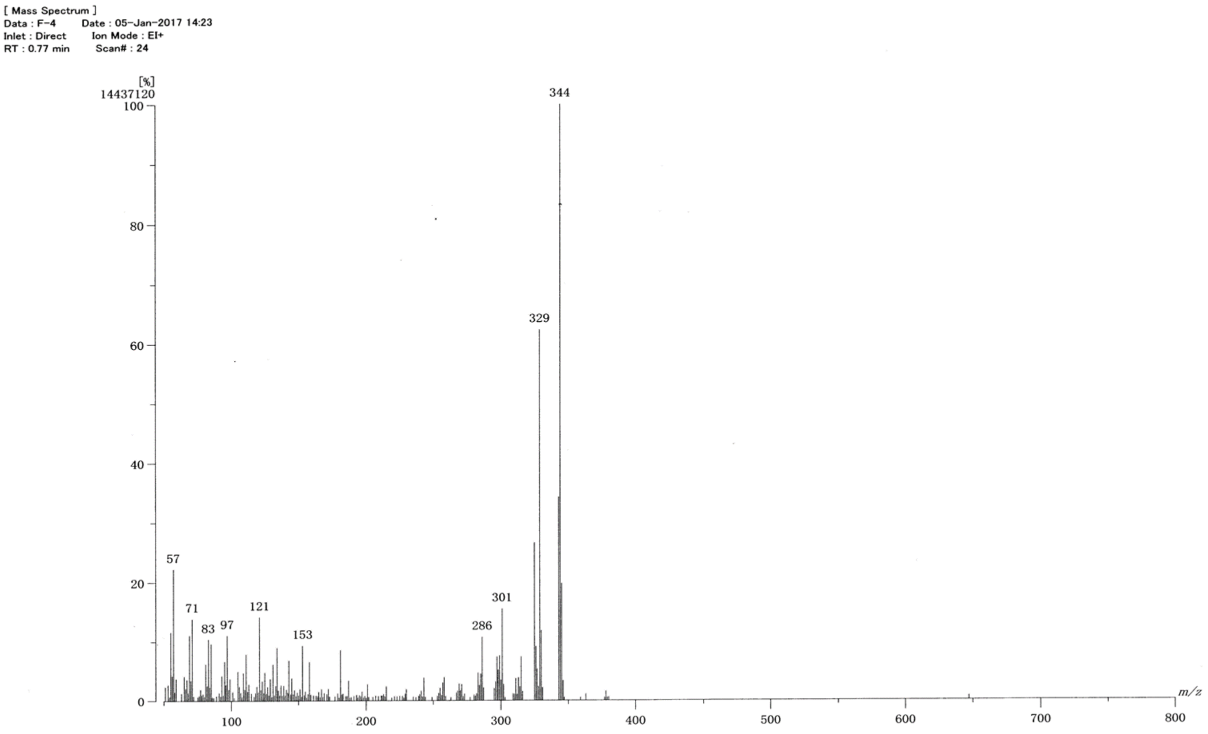

Figure 3. EIMS spectrum of compound **1**.

Data : F-4-HR Date : 05-Jan-2017 16:52
Instrument : MStation
Sample : -
Note : -
Inlet : Direct Ion Mode : EI+
RT : 1.77 min Scan# : 54
Elements : C 100/1, H 100/1, O 10/1
Mass Tolerance : 1000ppm, 3mmu if m/z > 3
Unsaturation (U.S.) : -0.5 - 20.0

	Observed m/z	Int%	Err[ppm / mmu]	U.S.	Composition
1	344.0891	36.56	-1.5 / -0.5	11.0	C18 H16 O7

Figure 4. HREIMS data of compound **1** ($[M]^+$).

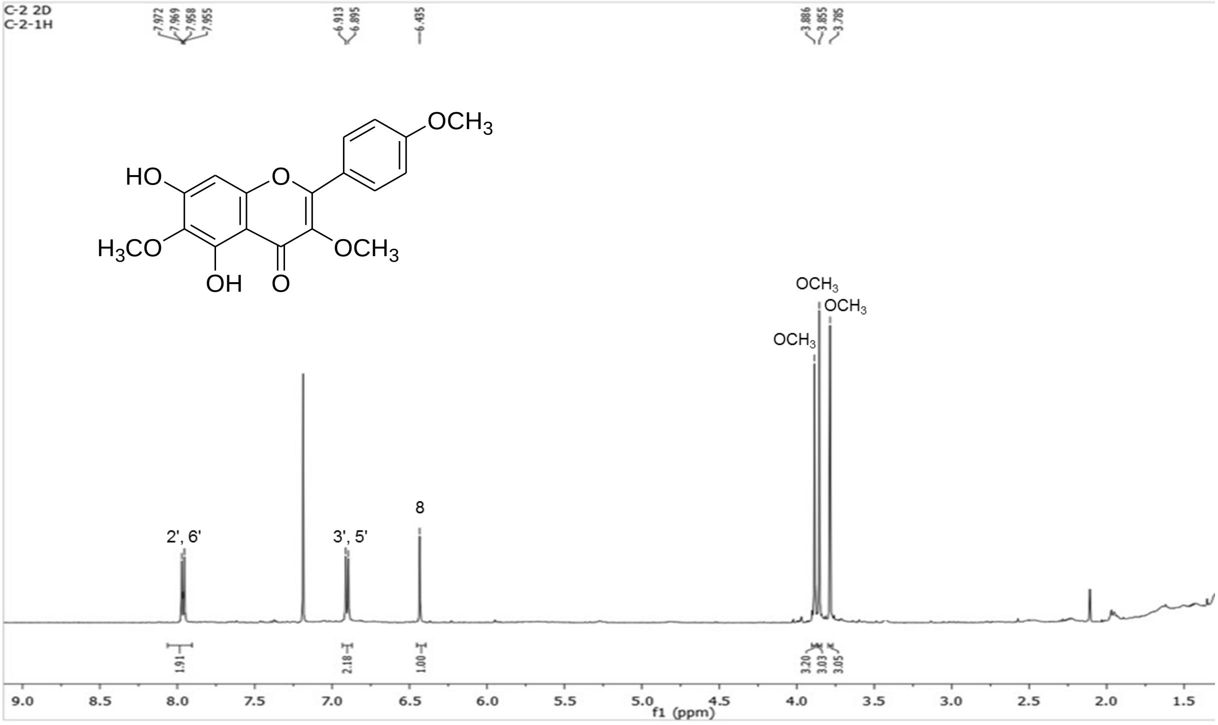

Figure 5. ^{1}H-NMR spectrum of compound **2** (500 MHz, $CDCl_3$).

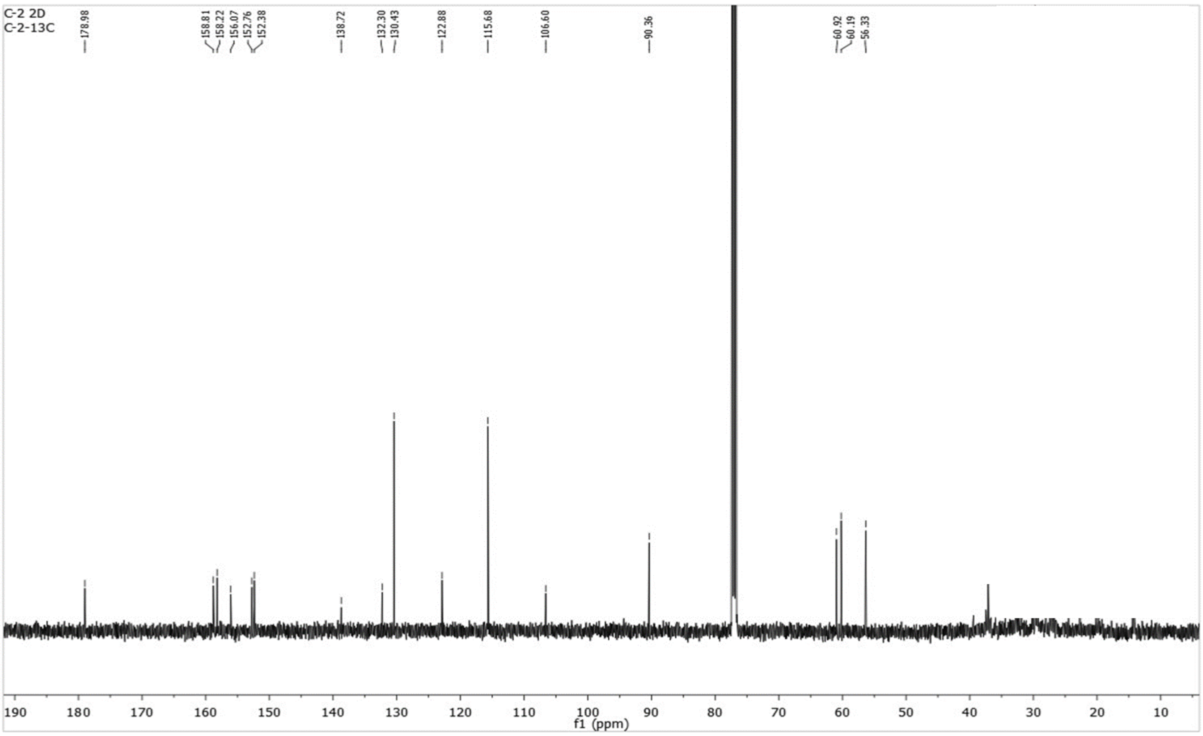

Figure 6. ^{13}C-NMR spectrum of compound **2** (125 MHz, $CDCl_3$).

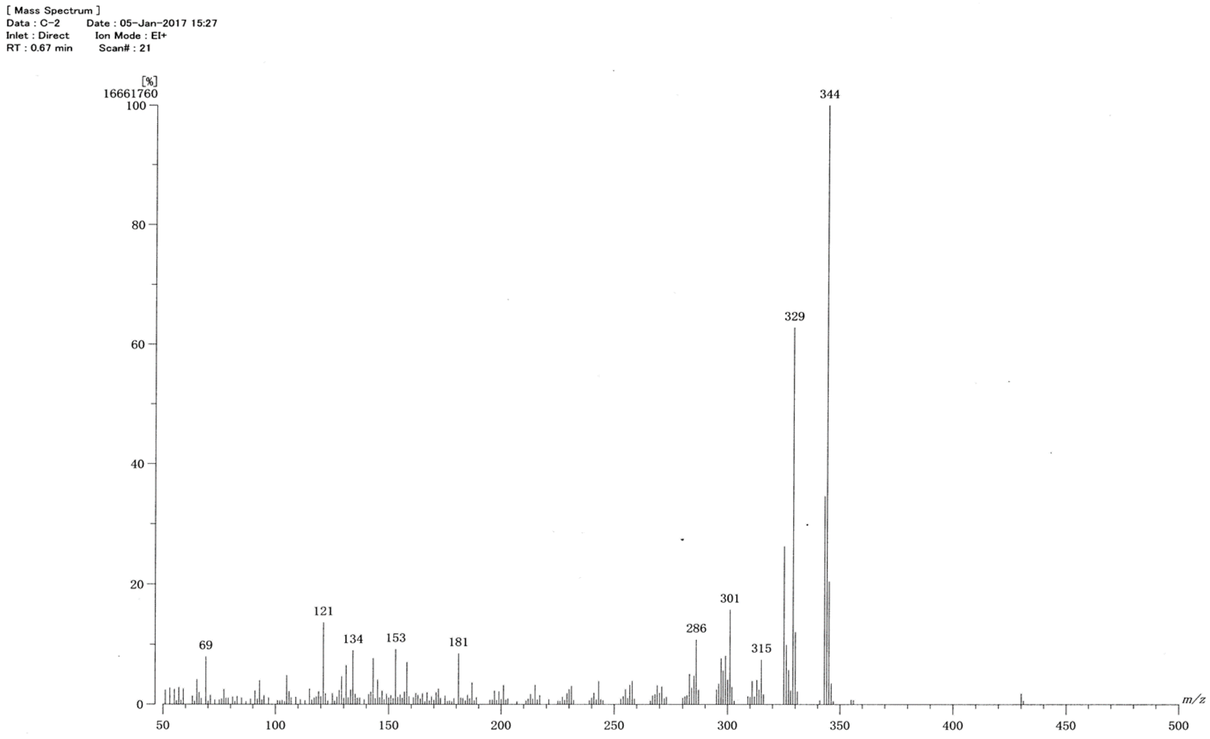

Figure 7. EIMS spectrum of compound **2**.

Data : C-2-HR Date : 06-Jan-2017 15:29
Instrument : MStation
Sample : -
Note : -
Inlet : Direct Ion Mode : EI+
RT : 1.14 min Scan# : 35
Elements : C 100/1, H 100/1, O 10/1
Mass Tolerance : 1000ppm, 3mmu if m/z > 3
Unsaturation (U.S.) : -0.5 - 20.0

	Observed m/z	Int%	Err[ppm / mmu]	U.S.	Composition
1	344.0898	100.00	+0.6 / +0.2	11.0	C18 H16 O7

Figure 8. HREIMS data of compound **2** ([M]$^{+}$).

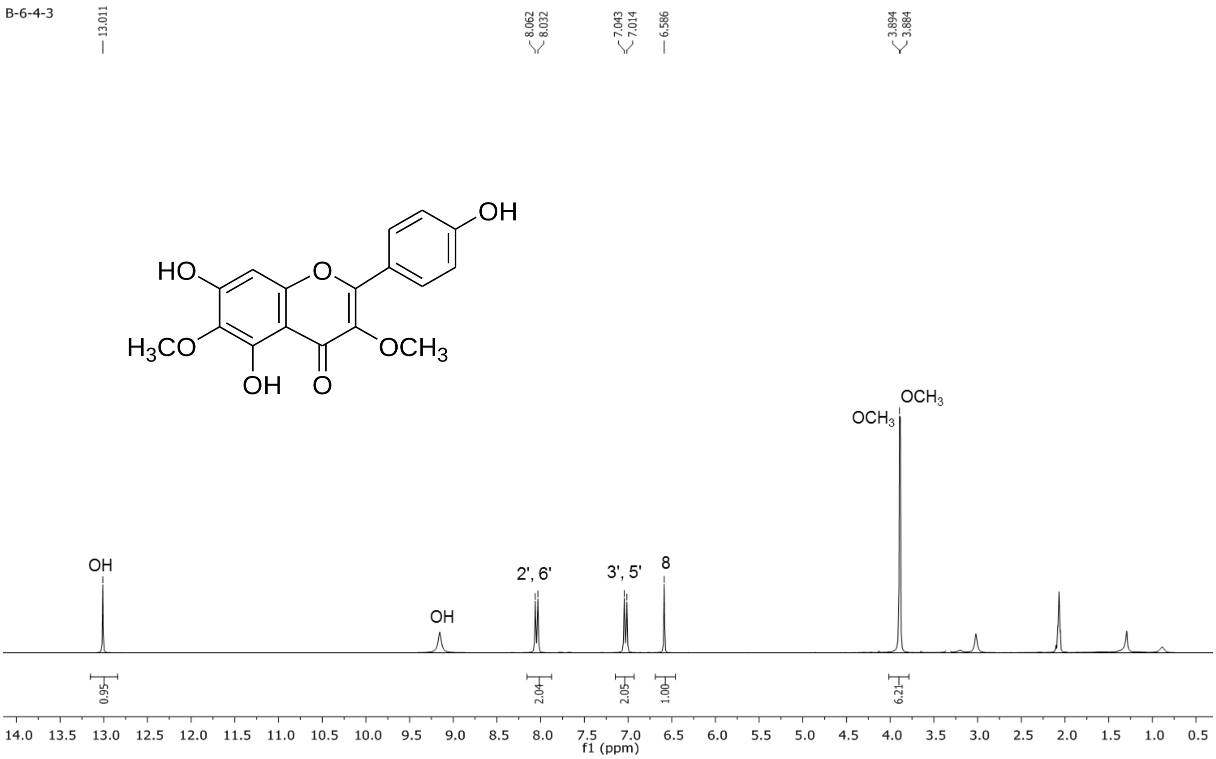

Figure 9. ^{1}H-NMR spectrum of compound **3** (500 MHz, Acetone).

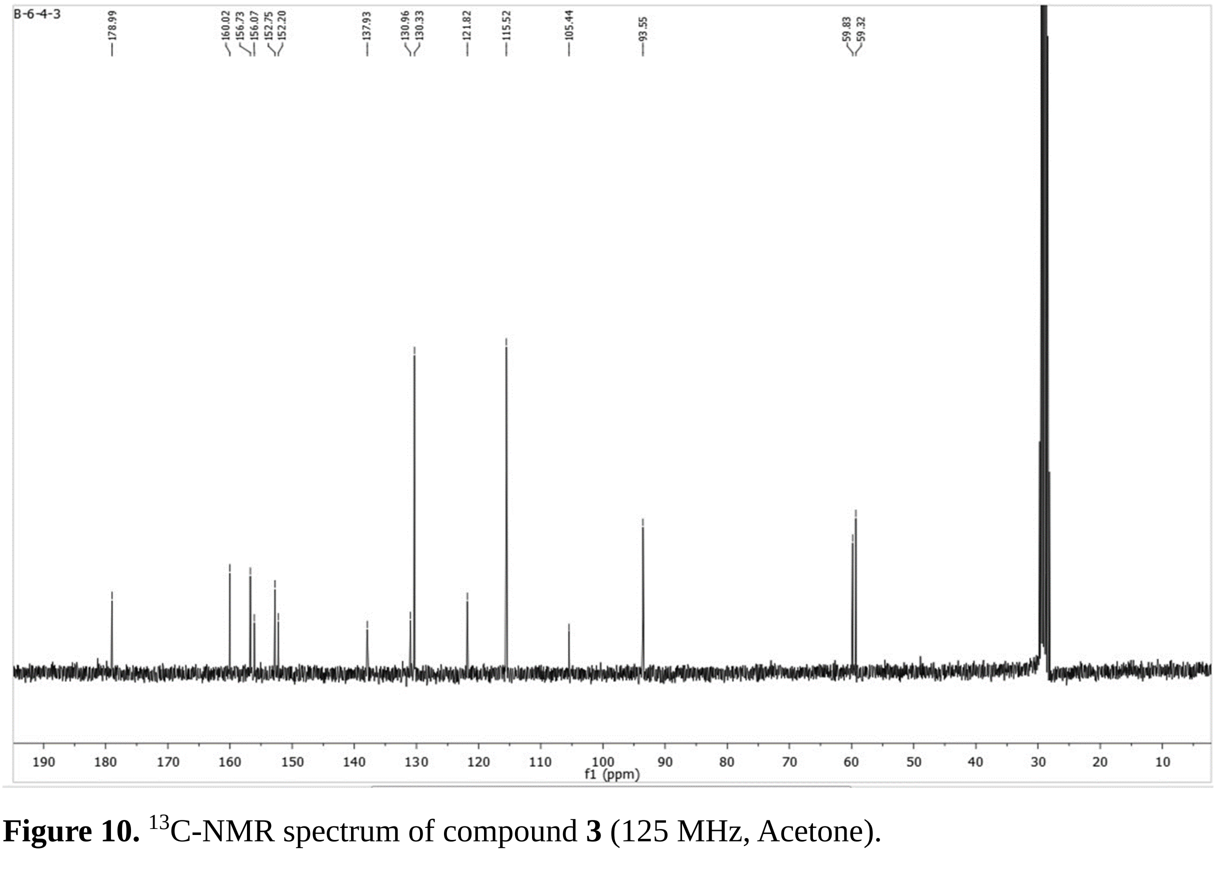

Figure 10. ^{13}C-NMR spectrum of compound **3** (125 MHz, Acetone).

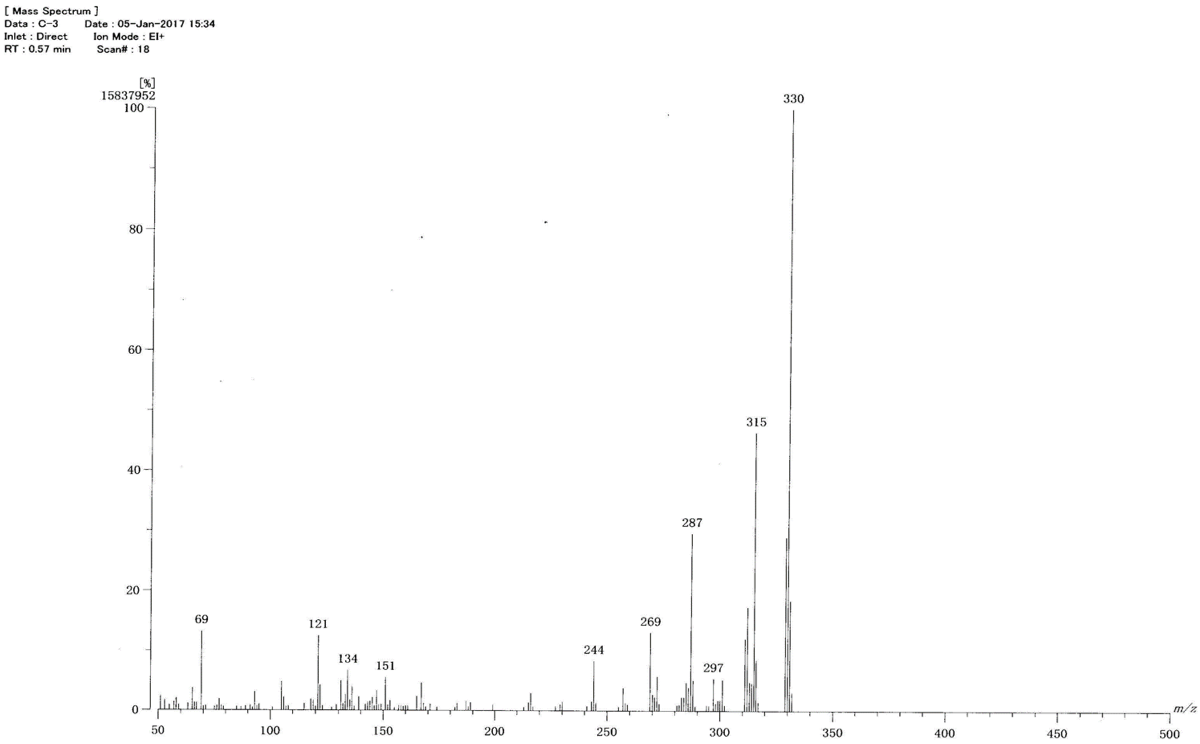

Figure 11. EIMS spectrum of compound **3**.

Data : C-3-HR Date : 06-Jan-2017 15:56
Instrument : MStation
Sample : -
Note : -
Inlet : Direct Ion Mode : EI+
RT : 0.20 min Scan# : 7
Elements : C 100/1, H 100/1, O 10/1
Mass Tolerance : 1000ppm, 3mmu if m/z > 3
Unsaturation (U.S.) : -0.5 - 20.0

	Observed m/z	Int%	Err[ppm / mmu]	U.S.	Composition
1	330.0747	100.00	+2.3 / +0.7	11.0	C17 H14 O7

Figure 12. HREIMS data of compound **3** ($[M]^+$).

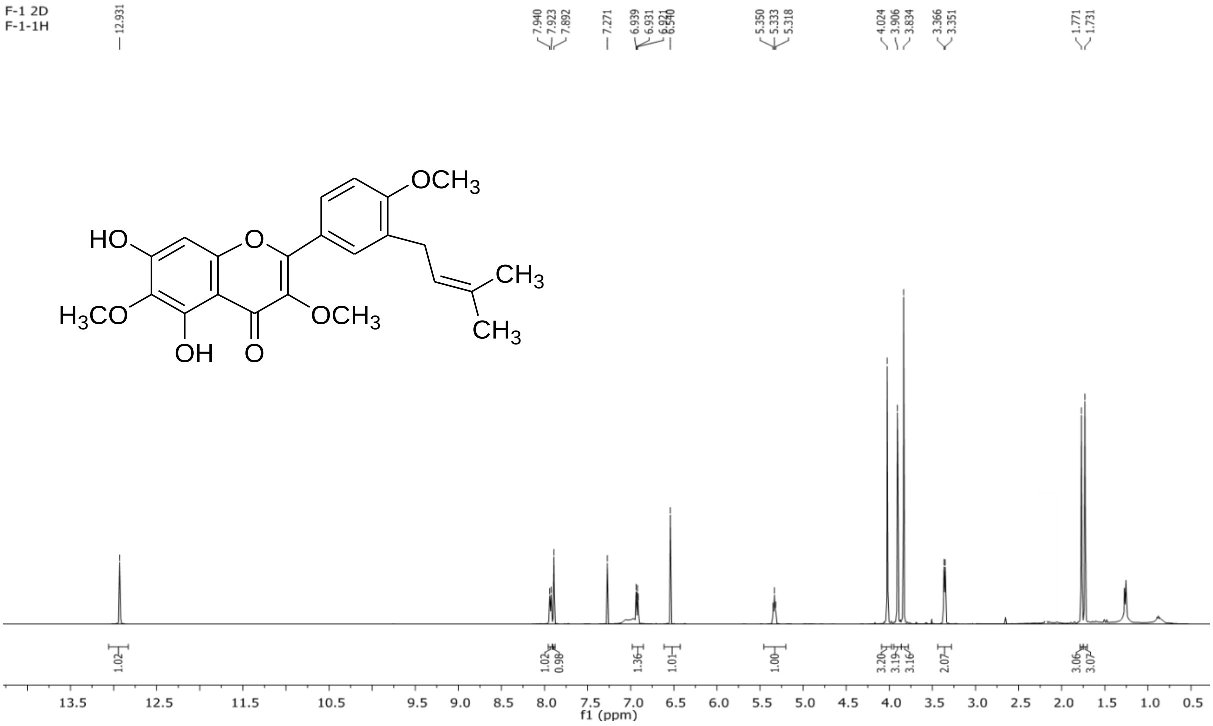

Figure 13. ^{1}H-NMR spectrum of compound **4** (500 MHz, $CDCl_3$).

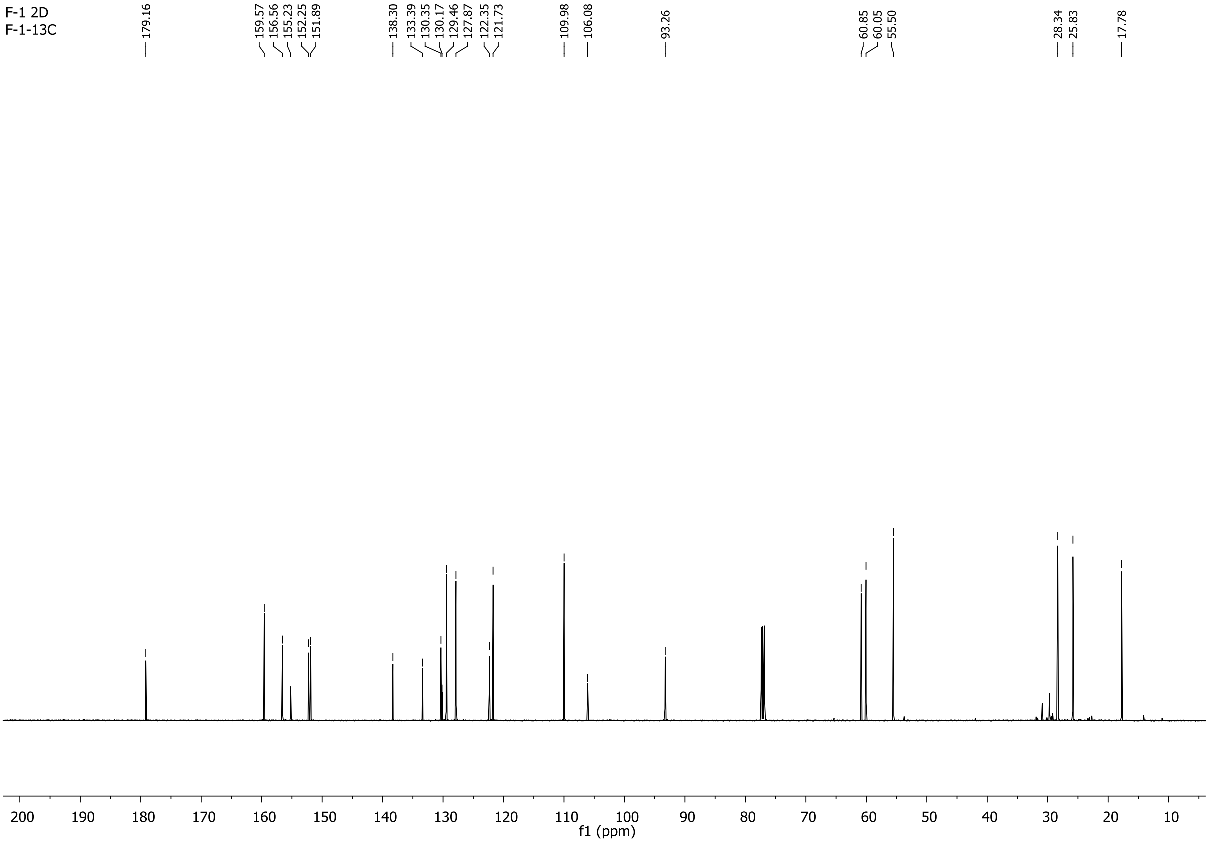

Figure 14. ^{13}C-NMR spectrum of compound **4** (125 MHz, $CDCl_3$).

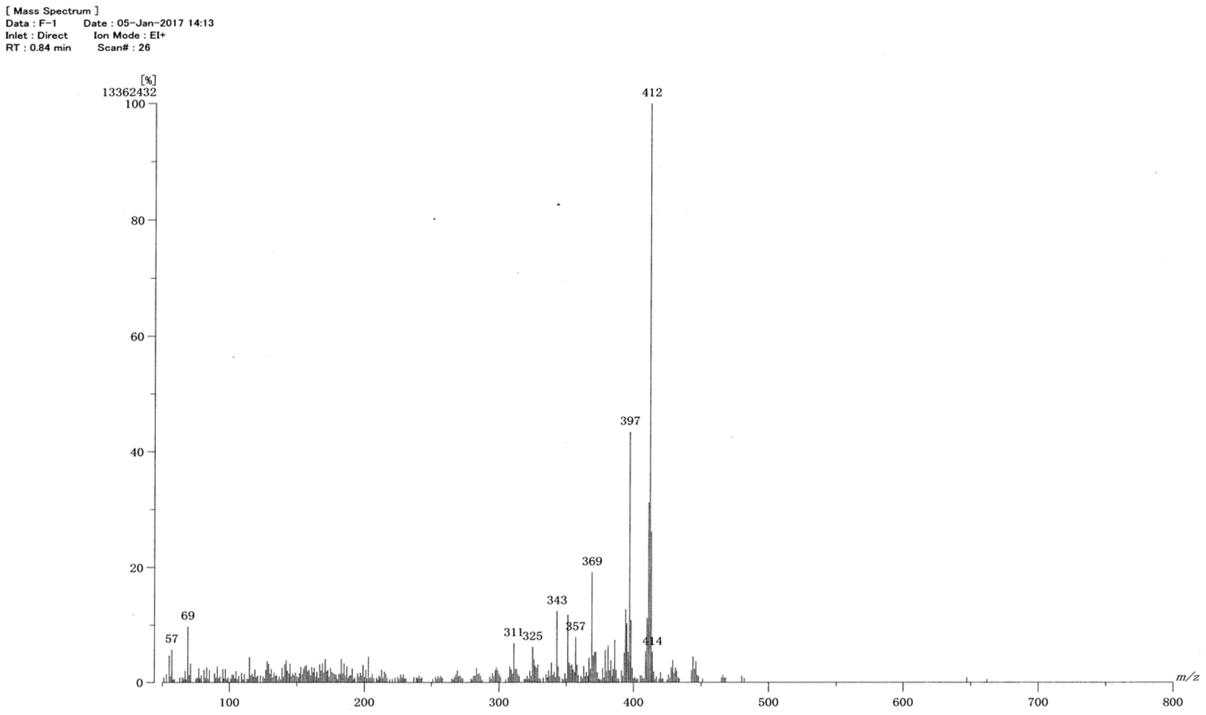

Figure 15. EIMS spectrum of compound **4**.

Data : F-1-HR Date : 05-Jan-2017 16:29
Instrument : MStation
Sample : -
Note : -
Inlet : Direct Ion Mode : EI+
RT : 1.07 min Scan# : 33
Elements : C 100/1, H 100/1, O 10/1
Mass Tolerance : 1000ppm, 3mmu if m/z > 3
Unsaturation (U.S.) : -0.5 - 20.0

	Observed m/z	Int%	Err[ppm / mmu]	U.S.	Composition
1	412.1520	100.00	-0.5 / -0.2	12.0	C23 H24 O7

Figure 16. HREIMS data of compound **4** ([M]$^+$).

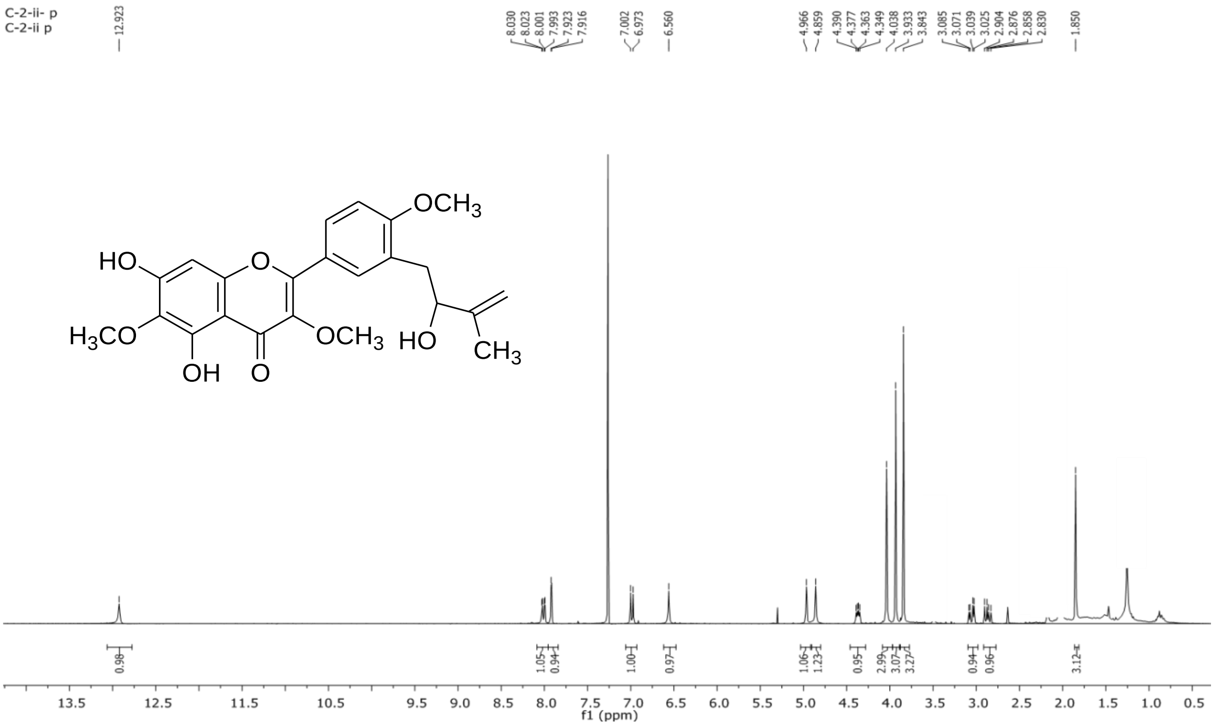

Figure 17. ^{1}H-NMR spectrum of compound **5** (500 MHz, $CDCl_3$).

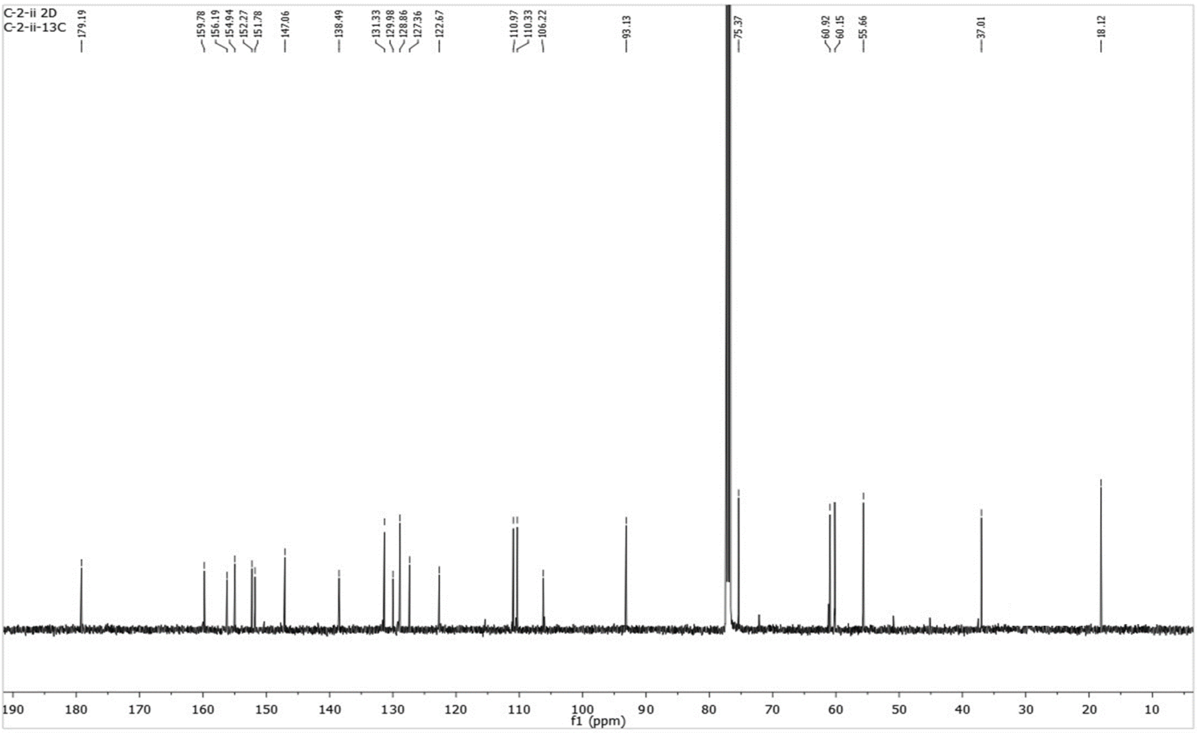

Figure 18. ^{13}C-NMR spectrum of compound **5** (125 MHz, $CDCl_3$).

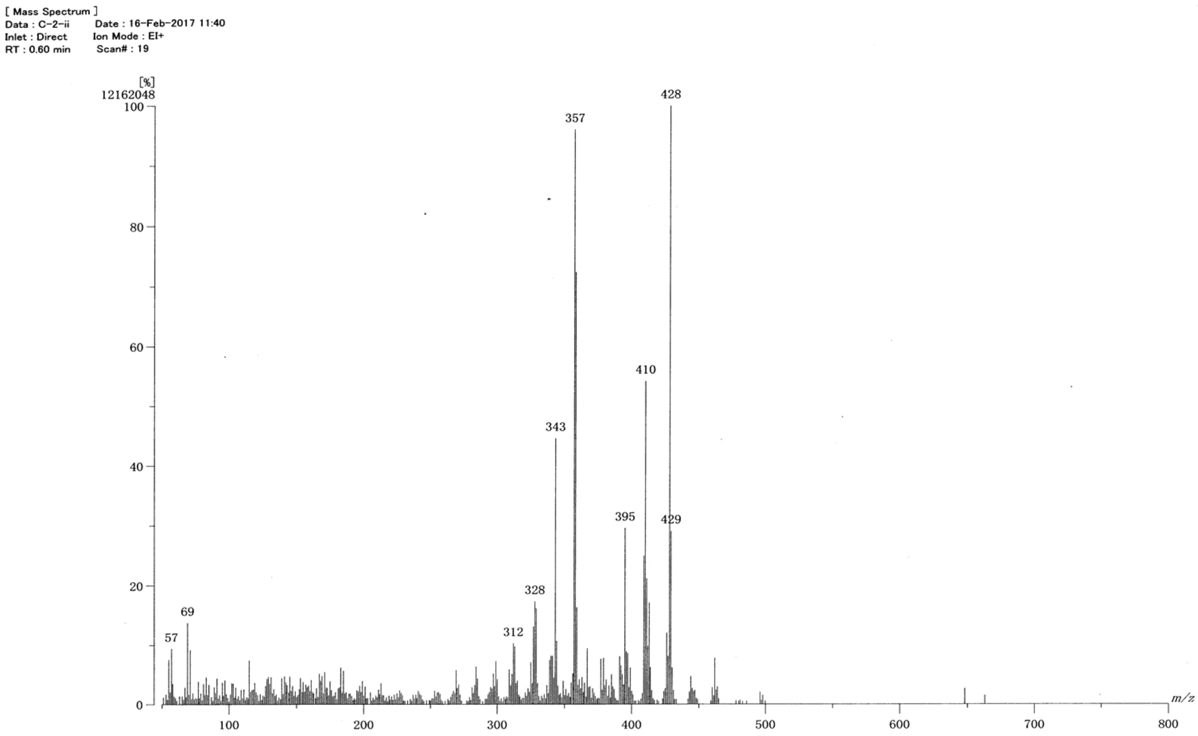

Figure 19. EIMS spectrum of compound **5**.

Data : C-2-ii-HR Date : 16-Feb-2017 11:56
Instrument : MStation
Sample : -
Note : -
Inlet : Direct Ion Mode : EI+
RT : 1.20 min Scan# : 37
Elements : C 100/1, H 100/1, O 10/1
Mass Tolerance : 1000ppm, 3mmu if m/z > 3
Unsaturation (U.S.) : -0.5 - 20.0

	Observed m/z	Int%	Err[ppm / mmu]	U.S.	Composition
1	428.1471	40.11	-0.0 / -0.0	12.0	C23 H24 O8

Figure 20. HREIMS data of compound **5** ($[M]^+$).

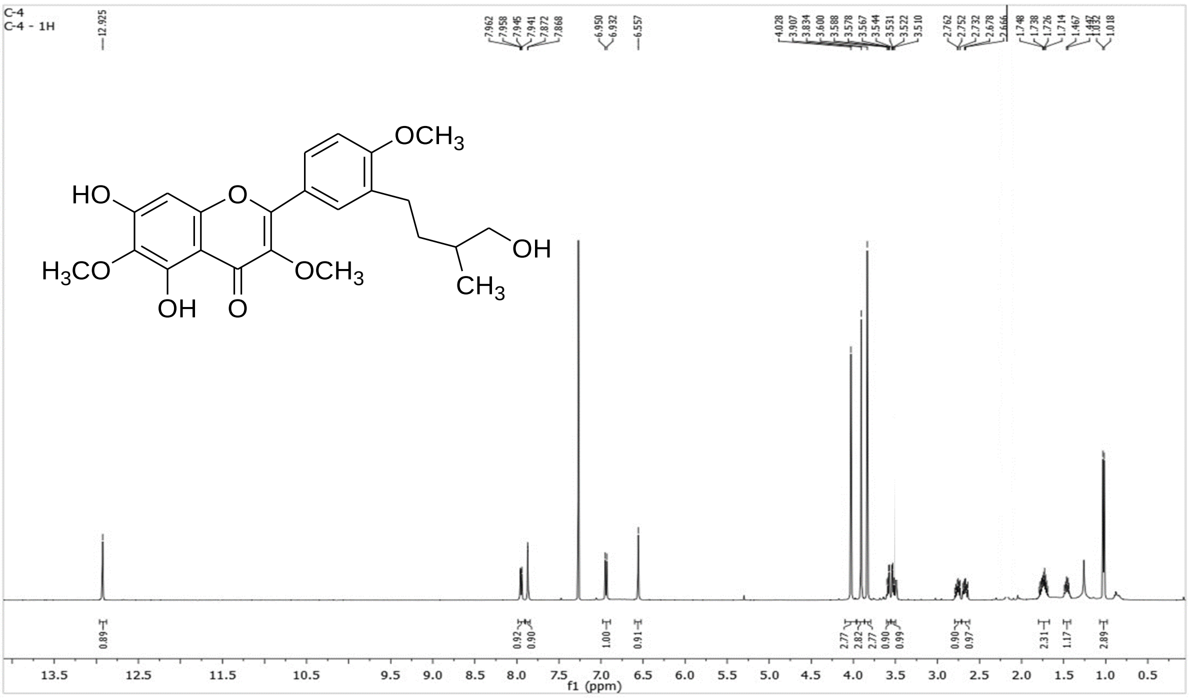

Figure 21. ^{1}H-NMR spectrum of compound **6** (500 MHz, $CDCl_3$).

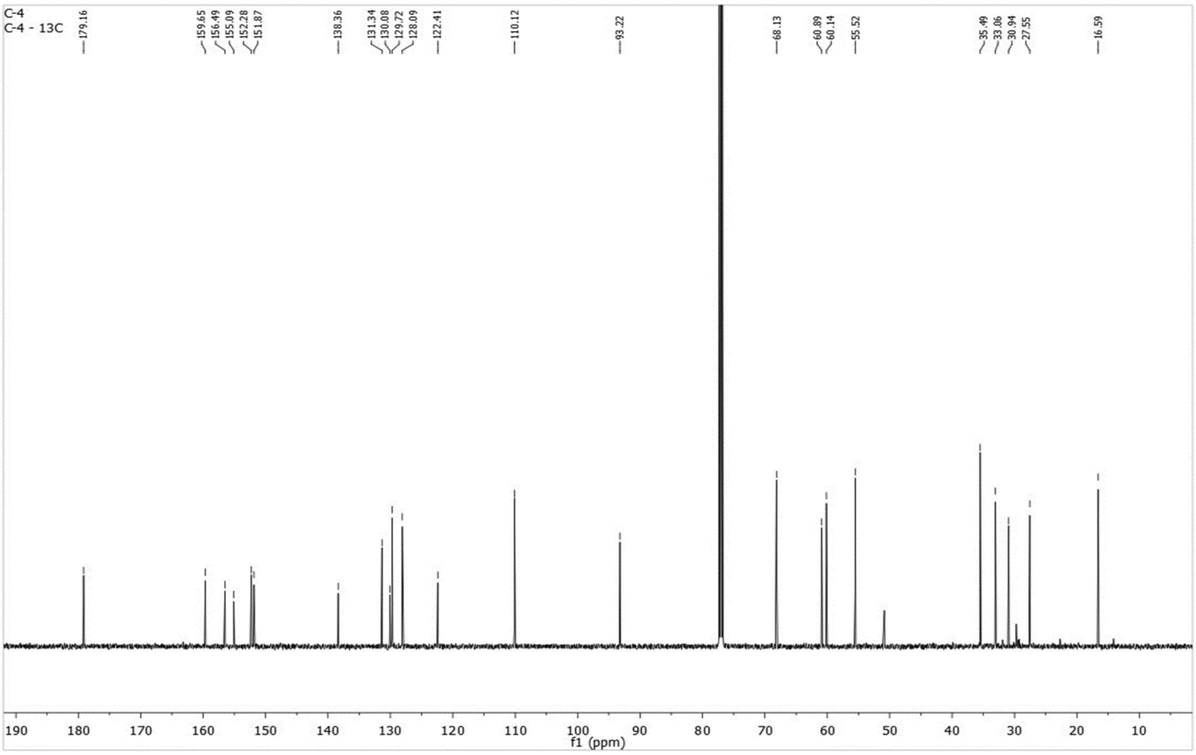

Figure 22. ^{13}C-NMR spectrum of compound **6** (125 MHz, $CDCl_3$).

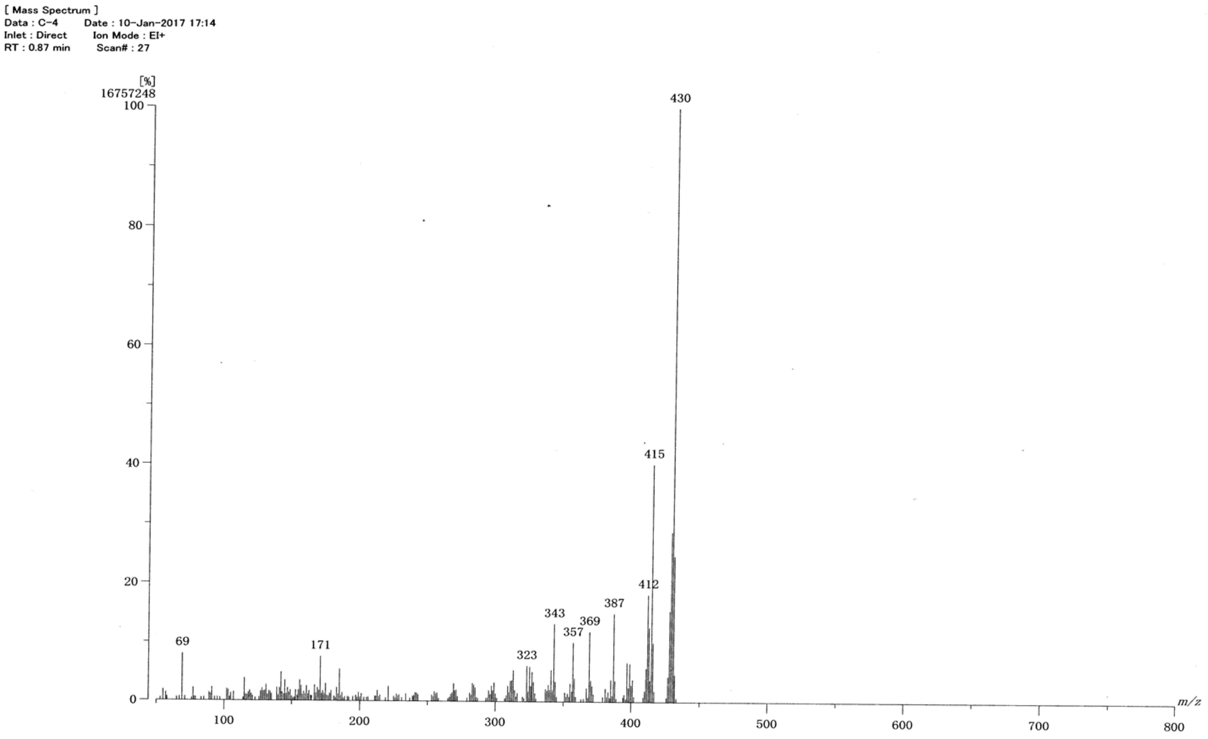

Figure 23. EIMS spectrum of compound **6**.

Data : C-4-HR Date : 25-Jan-2017 14:44
Instrument : MStation
Sample : -
Note : -
Inlet : Direct Ion Mode : EI+
RT : 0.94 min Scan# : 29
Elements : C 100/1, H 100/1, O 10/1
Mass Tolerance : 1000ppm, 3mmu if m/z > 3
Unsaturation (U.S.) : -0.5 - 20.0

	Observed m/z	Int%	Err[ppm / mmu]	U.S.	Composition
1	430.1629	100.00	+0.3 / +0.1	11.0	C23 H26 O8

Figure 24. HREIMS data of compound **6** ($[M]^+$).

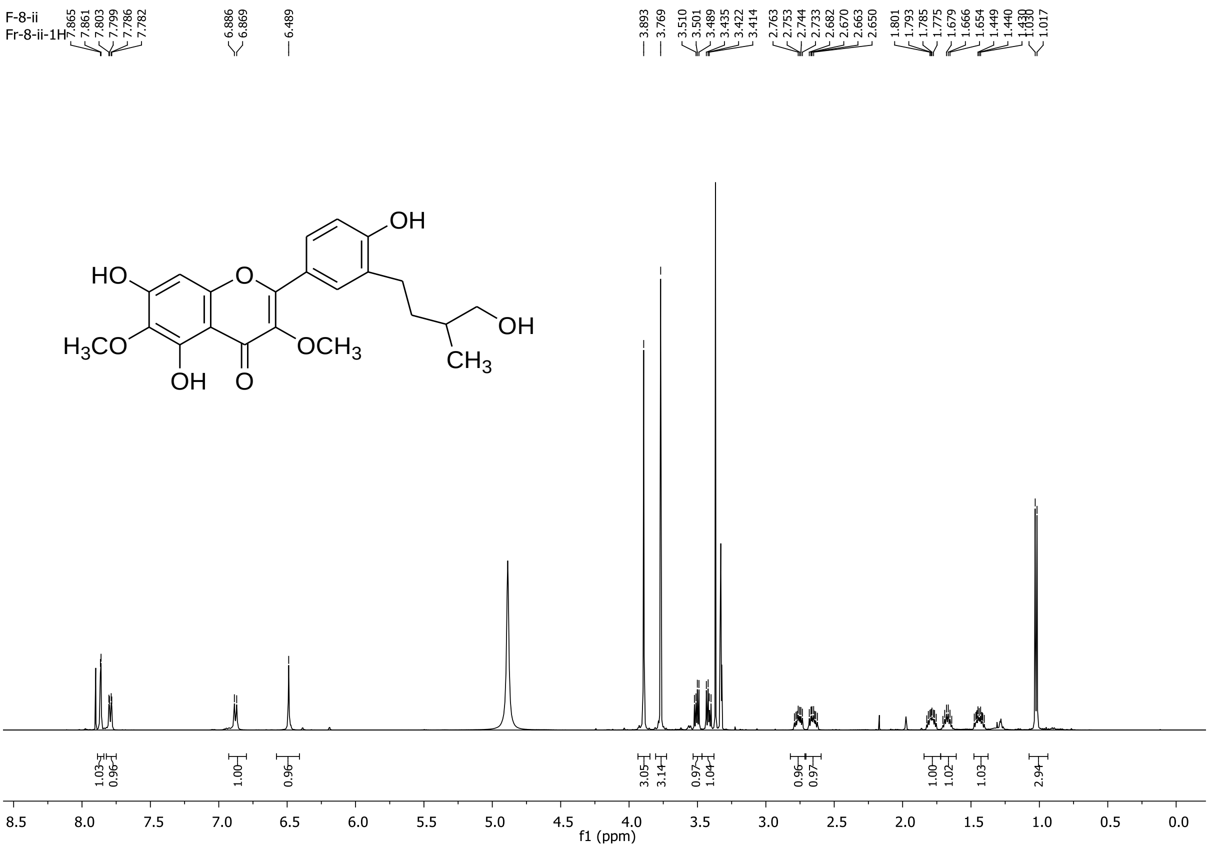

Figure 25. ^{1}H-NMR spectrum of compound **7** (500 MHz, CD_3OD).

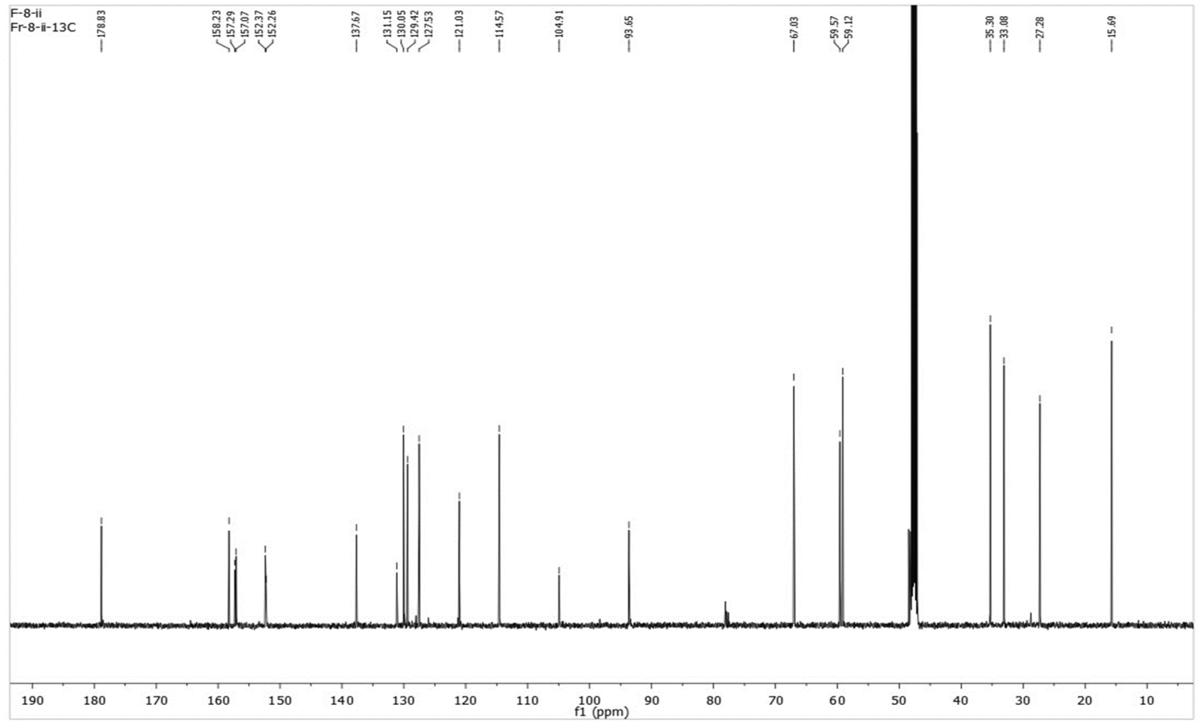

Figure 26. ^{13}C-NMR spectrum of compound **7** (125 MHz, CD_3OD).

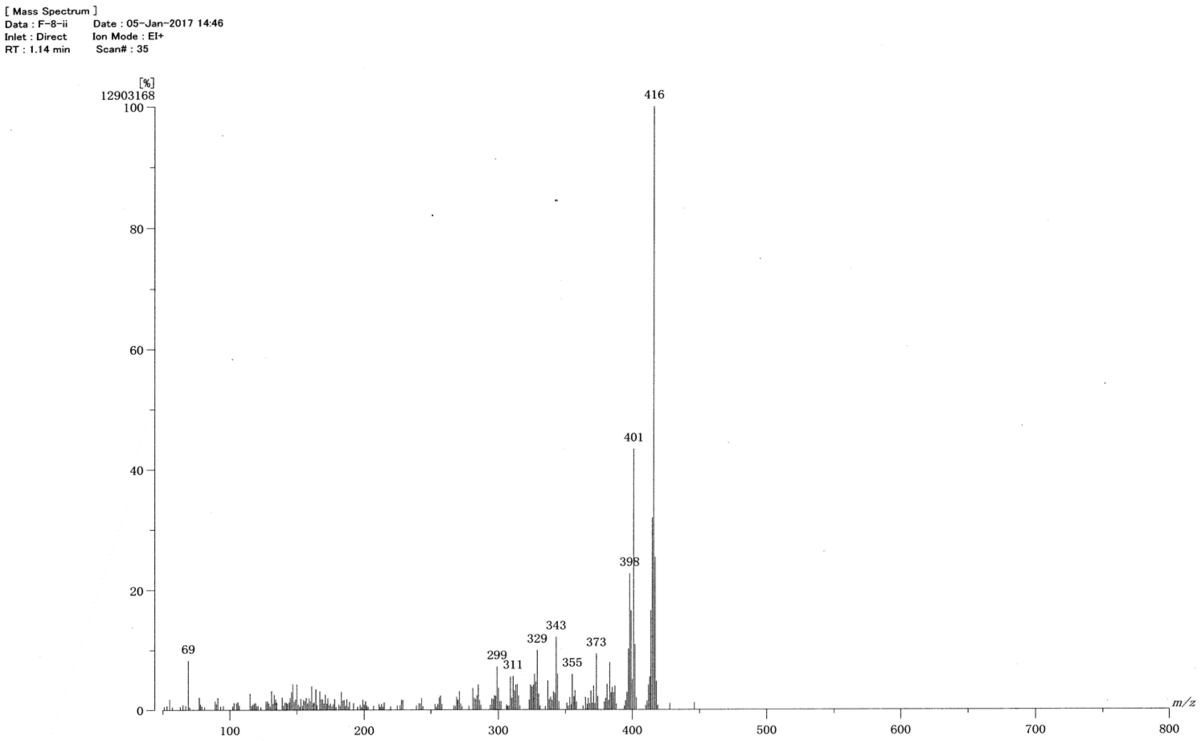

Figure 27. EIMS spectrum of compound **7**.

Data : F-8-ii-HR Date : 05-Jan-2017 17:08
Instrument : MStation
Sample : -
Note : -
Inlet : Direct Ion Mode : EI+
RT : 1.40 min Scan# : 43
Elements : C 100/1, H 100/1, O 10/1
Mass Tolerance : 1000ppm, 3mmu if m/z > 3
Unsaturation (U.S.) : -0.5 - 20.0

	Observed m/z	Int%	Err[ppm / mmu]	U.S.	Composition
1	416.1475	100.00	+0.9 / +0.4	11.0	C22 H24 O8

Figure 28. HREIMS data of compound **7** ($[M]^+$).

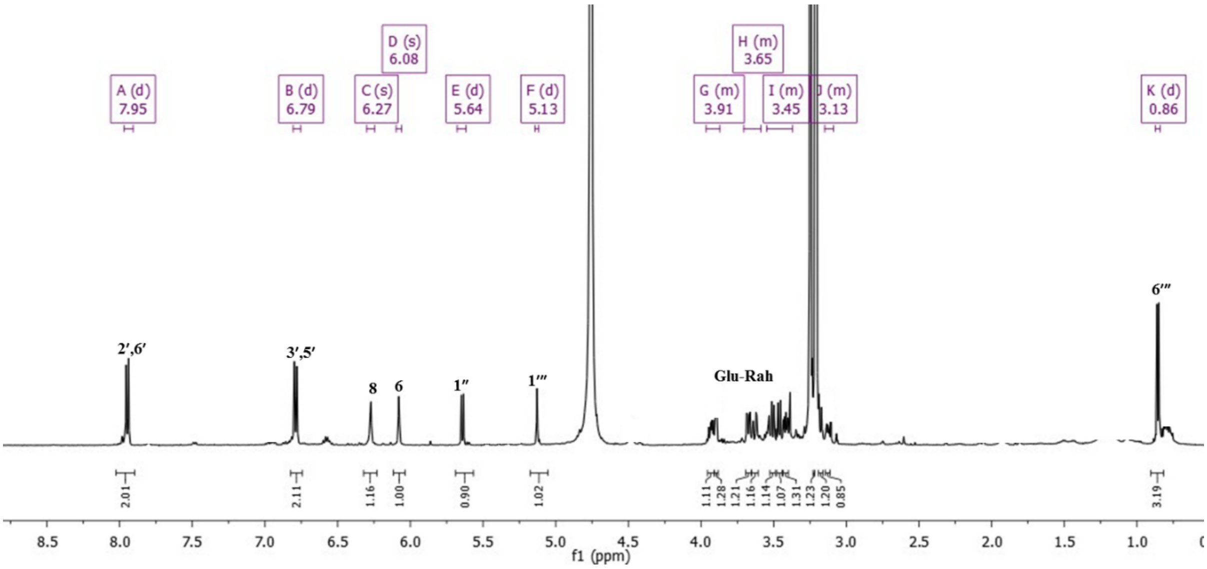

Figure 29. ^{1}H-NMR spectrum of compound **8** (500 MHz, CD_3OD).

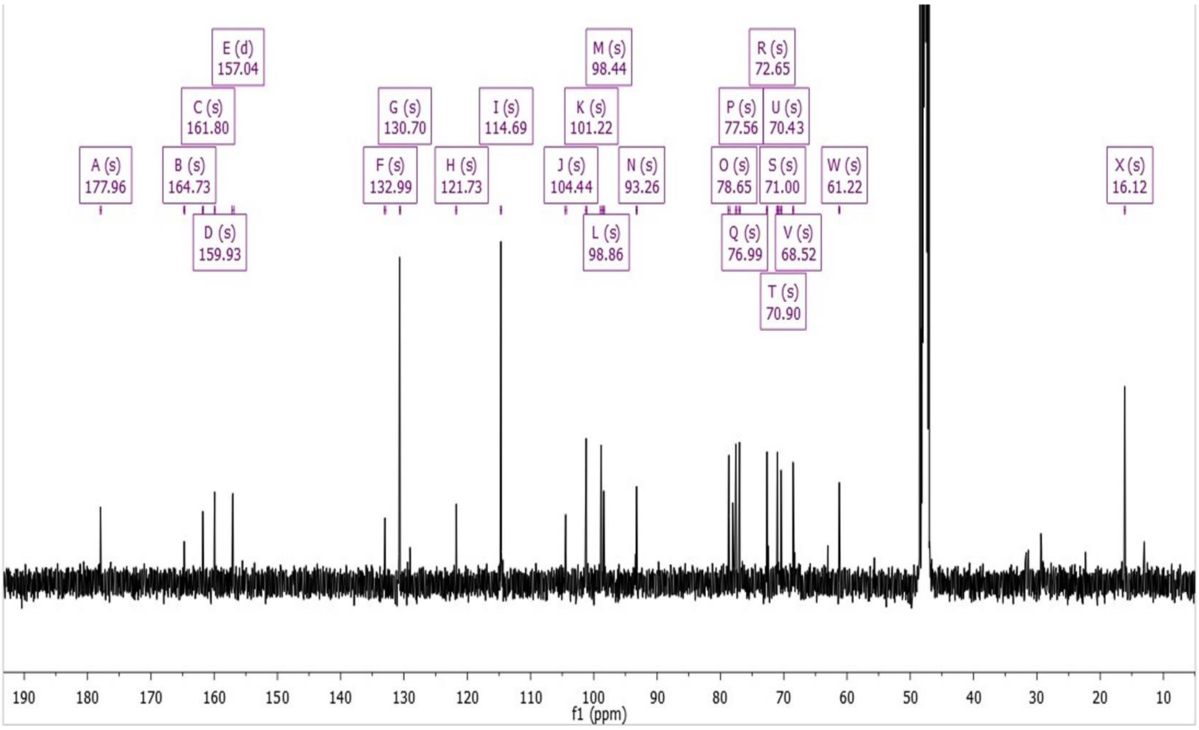

Figure 30. ^{13}C-NMR spectrum of compound **8** (125 MHz, CD_3OD).

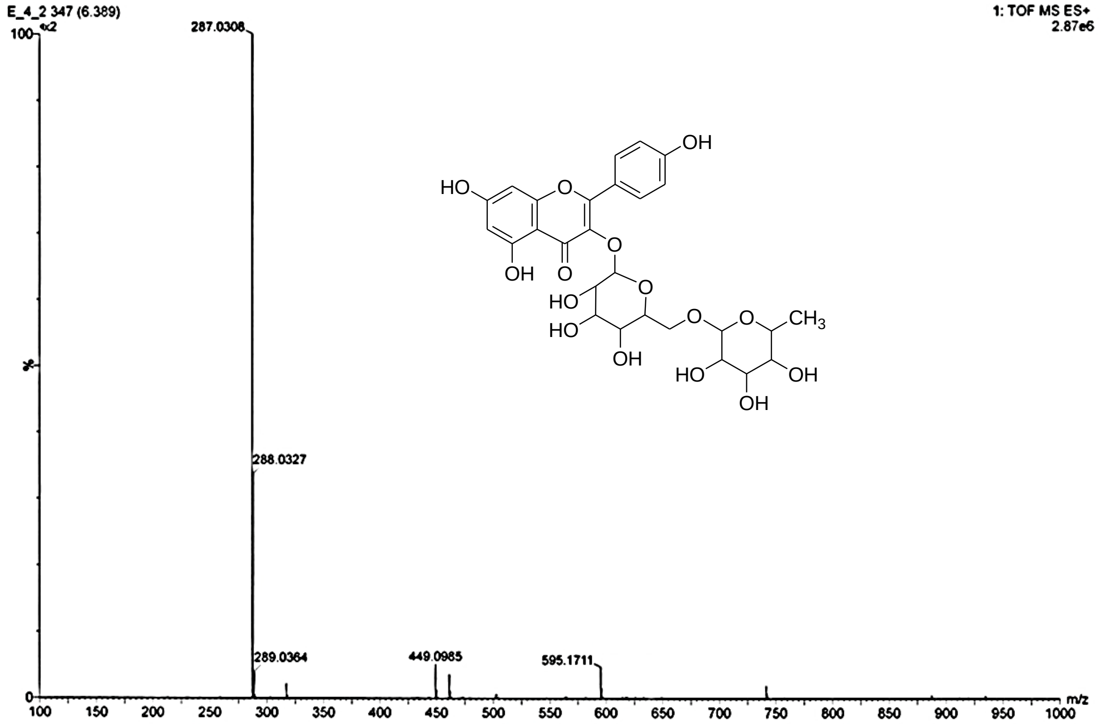

Figure 31. ESIMS spectrum of compound **8**.

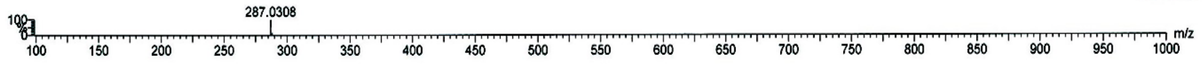

Elemental Composition Report

Single Mass Analysis
Tolerance = 10.0 mDa / DBE: min = -1.5, max = 50.0
Element prediction: Off

Monoisotopic Mass, Even Electron Ions
136 formula(e) evaluated with 4 results within limits (all results (up to 1000) for each mass)
Elements Used:
C: 0-50 H: 0-50 O: 0-50
E_4_2 348 (6.406)
1: TOF MS ES+

Minimum:				-1.5	
Maximum:		10.0	10.0	50.0	
Mass	Calc. Mass	mDa	PPM	DBE	Formula
595.1711	595.1722	-1.1	-1.8	3.5	C20 H35 O20
	595.1757	-4.6	-7.7	25.5	C38 H27 O7
	595.1698	1.3	2.2	34.5	C45 H23 O2
	595.1663	4.8	8.1	12.5	C27 H31 O15

Figure 32. HRESIMS data of compound **8** ($[M+H]^+$).

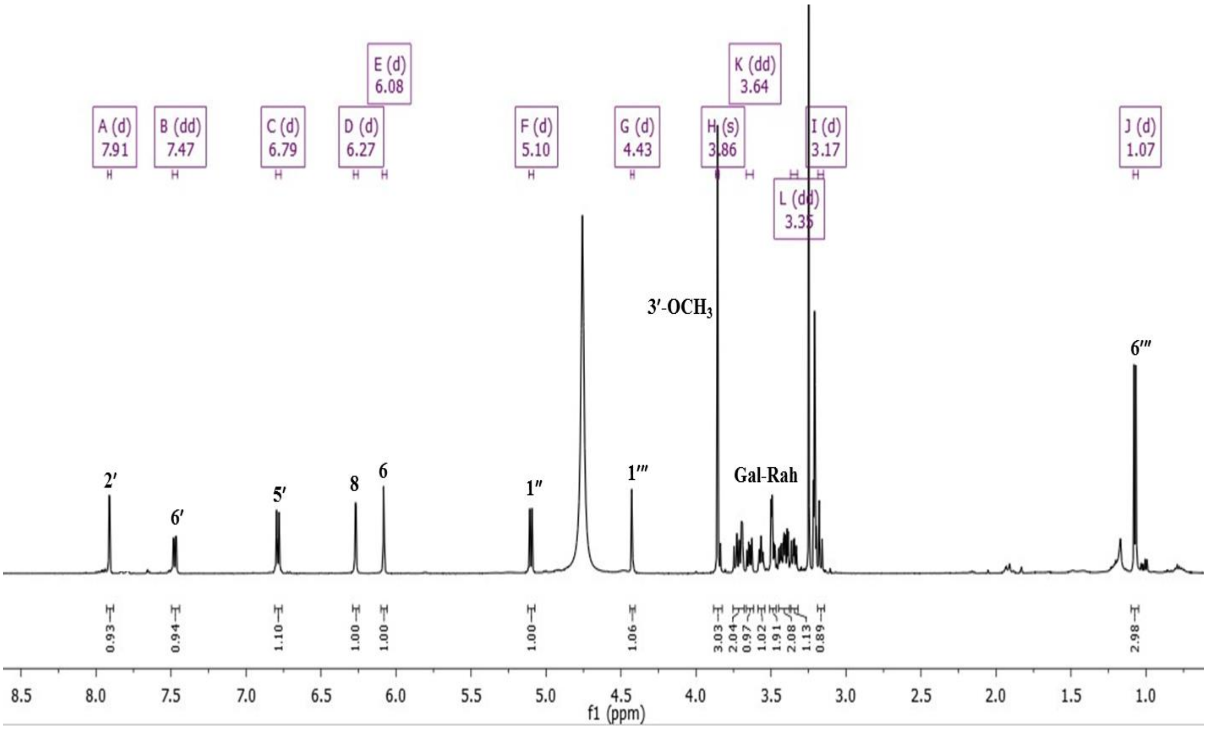

Figure 33. ^{1}H-NMR spectrum of compound **9** (500 MHz, CD_3OD).

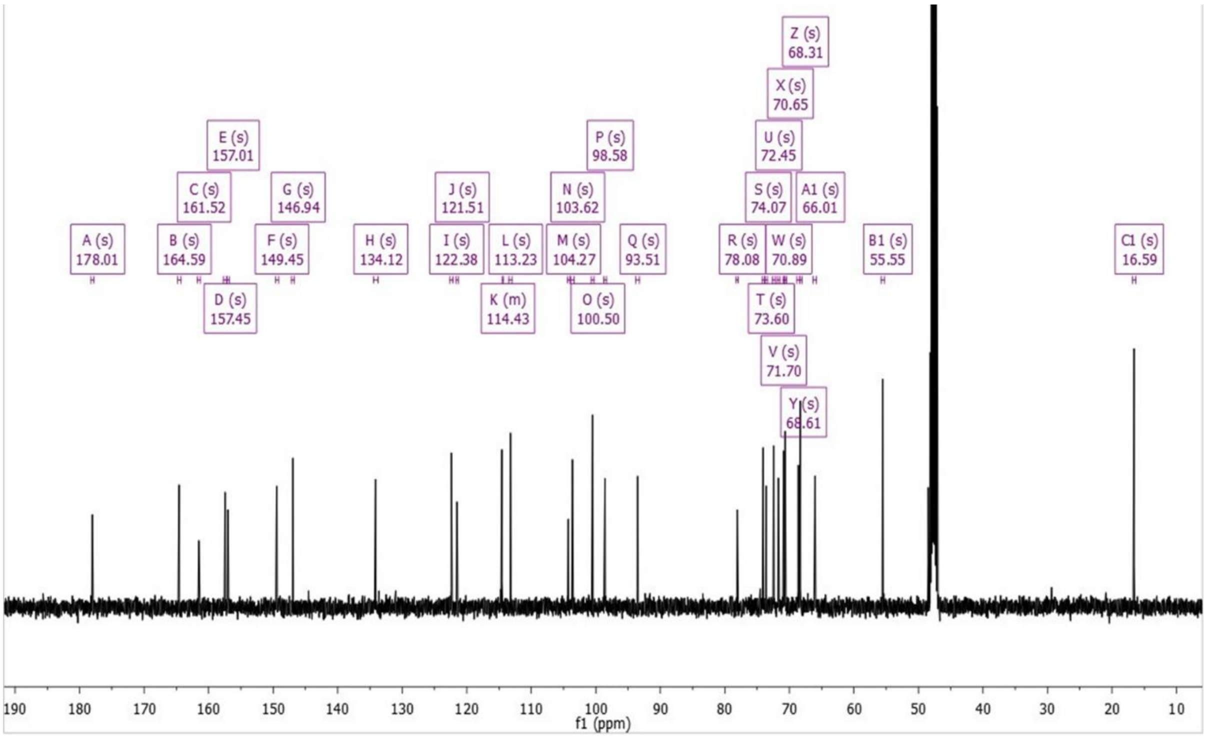

Figure 34. ^{13}C-NMR spectrum of compound **9** (125 MHz, CD_3OD).

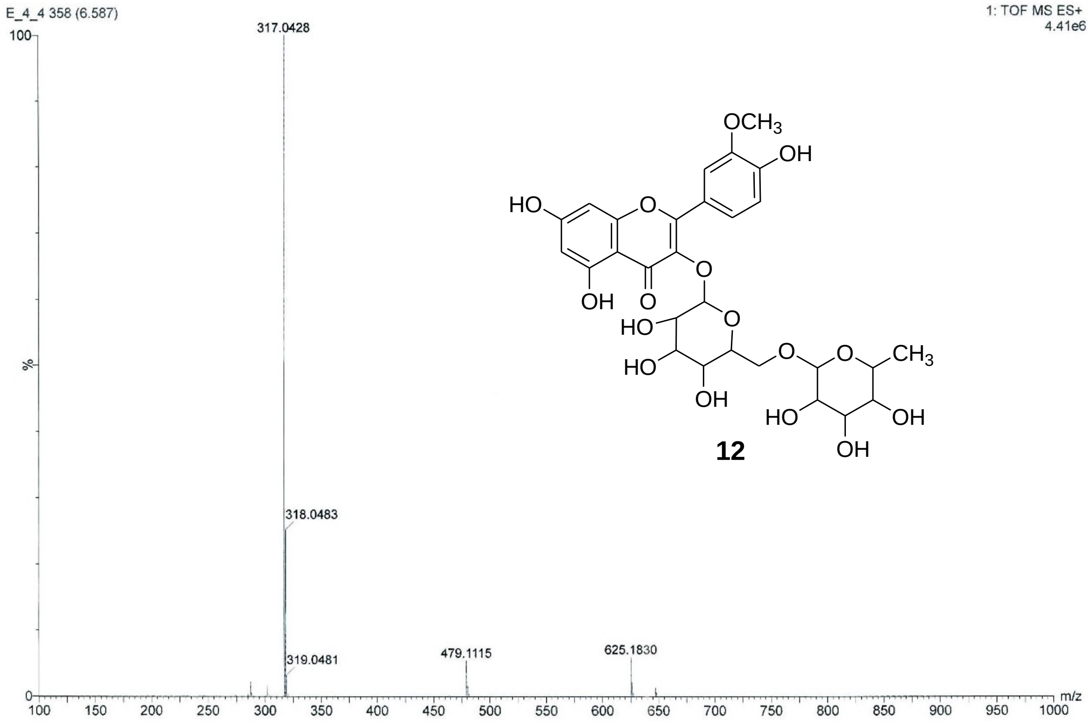

Figure 35. ESIMS spectrum of compound **9**.

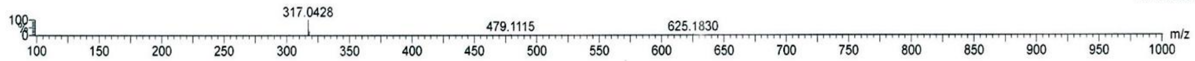

Elemental Composition Report

Single Mass Analysis
Tolerance = 10.0 mDa / DBE: min = -1.5, max = 50.0
Element prediction: Off

Monoisotopic Mass, Even Electron Ions
140 formula(e) evaluated with 5 results within limits (all results (up to 1000) for each mass)
Elements Used:
C: 0-50 H: 0-50 O: 0-50
E_4_4 358 (6.587)
1: TOF MS ES+

Minimum:				-1.5	
Maximum:		10.0	10.0	50.0	
Mass	Calc. Mass	mDa	PPM	DBE	Formula
625.1830	625.1827	0.3	0.5	3.5	C21 H37 O21
	625.1921	-9.1	-14.6	16.5	C32 H33 O13
	625.1862	-3.2	-5.1	25.5	C39 H29 O8
	625.1804	2.6	4.2	34.5	C46 H25 O3
	625.1769	6.1	9.8	12.5	C28 H33 O16

Figure 36. HRESIMS data of compound **9** ($[M+H]^+$).

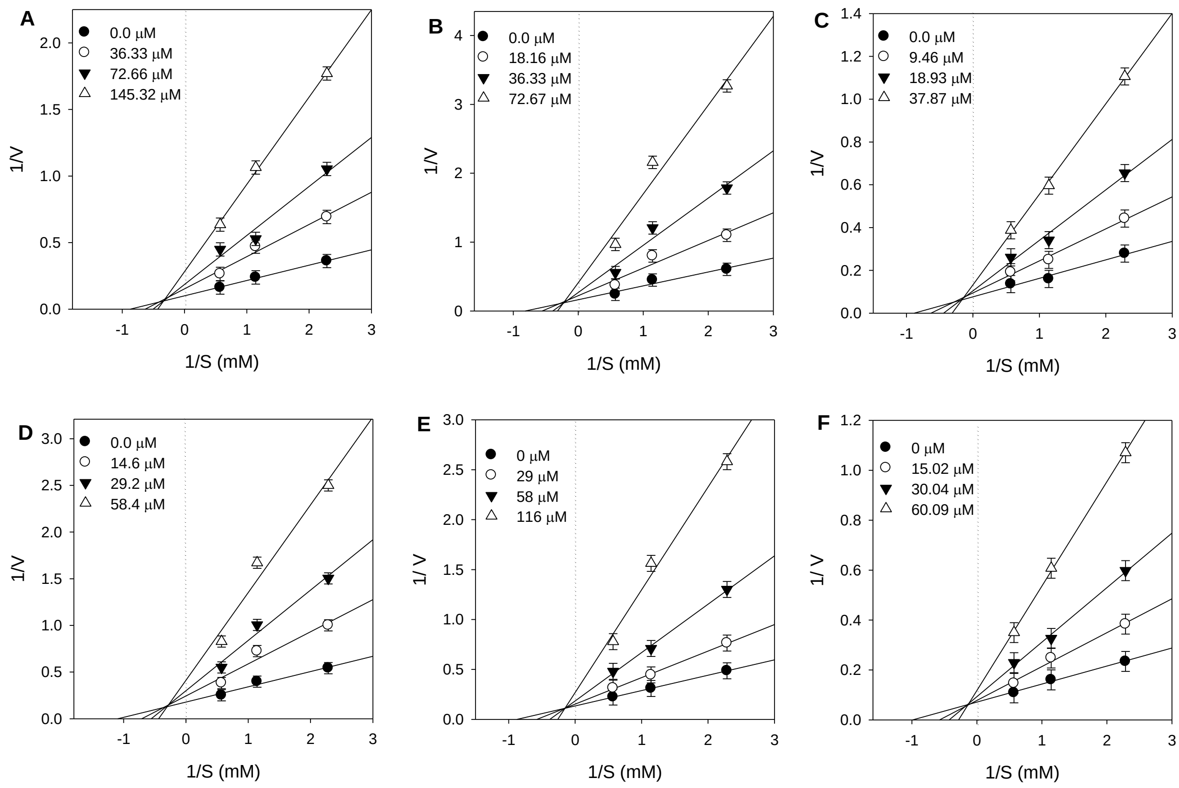

Figure 37. Lineweaver-Burk plots for PTP1B inhibition by Compound **1** (A), **2** (B), **3** (C), **5** (D), **6** (E), **7** (F).

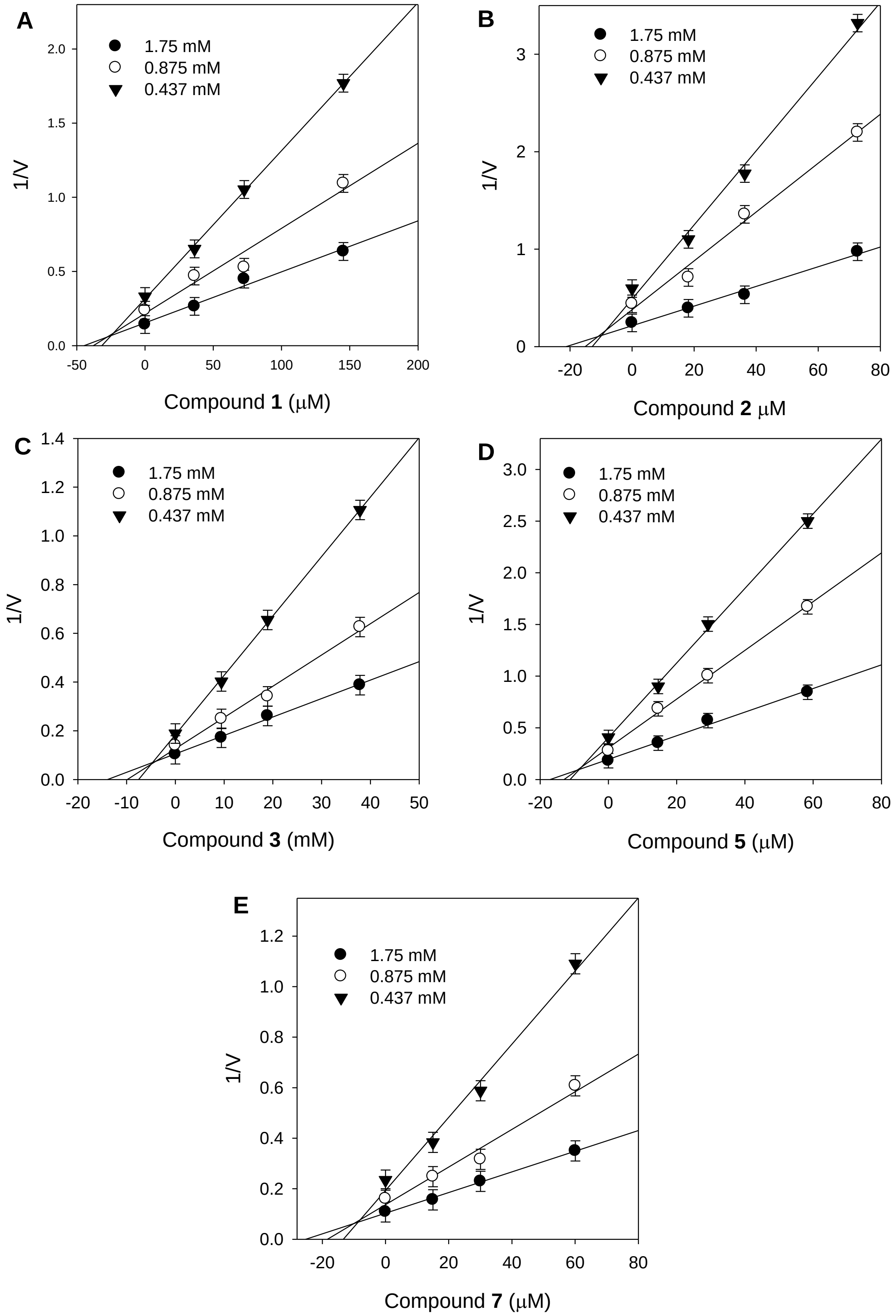

Figure 38. Dixon plots for PTP1B inhibition by Compound **1** (A), **2** (B), **3** (C), **5** (D), and **7** (E) respectively.
